# Electrohydrogenation of Benzonitrile into Benzylamine under Mild Aqueous Conditions

**DOI:** 10.1021/acssuschemeng.5c02168

**Published:** 2025-06-05

**Authors:** Jose Solera-Rojas, Carles Forés, Guillem Beltrán-Gargallo, Francisco Fabregat-Santiago, José A. Mata, Carmen Mejuto, Elena Mas-Marzá

**Affiliations:** Institute of Advanced Materials (INAM), 16748 Universitat Jaume I, 12006 Castello, Spain

**Keywords:** electrochemical hydrogenation, benzonitrile reduction, electrocatalysis, copper-based electrodes, copper−silver-based electrodes

## Abstract

The electrohydrogenation of benzonitrile to benzylamine was investigated by using copper and copper–silver electrodes under mild conditions and moderate current density. In this study, we optimized several reaction parameters, including solvent, current density applied, electrolyte effect, and substrate concentration. The results reveal that the best performance in terms of yield, conversion, and faradaic efficiency was obtained at −20 mA·cm^–2^, using copper–silver electrodes, at neutral pH (0.5 M KCl). Our study highlights the potential of electrochemistry to enhance reduction reactions by electrohydrogenation using only water as the proton source and demonstrates the potential of copper–silver electrodes for efficient and environmentally friendly electrochemical hydrogenation at neutral pH. Additionally, our research aims to emphasize the capability of electrocatalysis to undergo reversible electrohydrogenation/dehydrogenation transformations using the nitrile/amine pair as a promising candidate in the field of hydrogen storage as liquid organic hydrogen carriers. This study not only provides insights into the possibility of sustainable hydrogenation processes but also contributes to further exploration of electrocatalytic systems for scalable, efficient, and environmentally friendly hydrogen storage solutions, such as the nitrile/amine pair.

## Graphical abstract

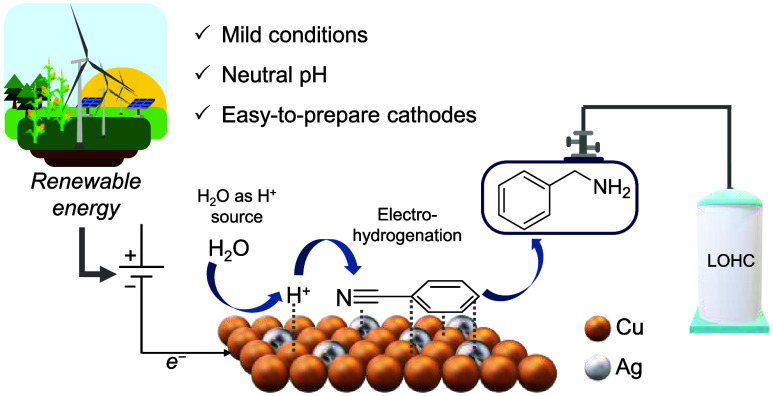

## Introduction

Due to the rise in global energy consumption, one of the critical challenges facing our society is the development of sustainable and clean energy systems.^1^ The use of fossil fuels, a major contributor to climate change, demands a transition to more carbon-neutral alternatives like biofuels and other renewable resources.^2,3^

Currently, hydrogen is considered a promising nonpolluting energy vector due to its high energy density and low activation energy, serving as a medium for storing and transporting energy without producing harmful environmental emissions when used. However, molecular hydrogen gas has significant limitations in terms of storage, transport, distribution, and handling with the corresponding associated safety requirements. All of these impediments make it difficult to use molecular hydrogen gas as an energy vector or even to carry out some industrial applications. To address these challenges, liquid organic hydrogen carriers (LOHCs) have emerged as a promising alternative for hydrogen storage and release via hydrogenation/dehydrogenation chemical reactions.^4,5^ Several organic molecules with highly oxidizable and/or reducible functional groups, such as alcohols,^6,7^ amines,^8–10^ ketones,^11^ or alkenes,^12^ in aromatic or aliphatic skeletons, have been postulated as promising LOHCs.

In the particular case of the LOHC pair amine/nitrile, the integration of electrocatalysis for the hydrogenation/dehydrogenation processes represents a significant advancement in the field of energy and hydrogen storage, where the nitrile and amine groups can serve as reactive sites for hydrogen storage and release, respectively. This approach allows hydrogen storage for using it as an energy vector in a safe and suitable manner in the liquid state without the need of harsh and strict requirements of transport control. Hydrogen can be released through electrodehydrogenation of amines into nitriles and subsequently recovered by electrohydrogenation of nitriles back into amines by using water as a source of protons. In addition, the use of nitriles as substrates for hydrogenation reactions yields primary amines, which are valuable compounds for their use as intermediates and precursors in the synthesis of agrochemicals, dyes, pigments, polymers, and pharmaceuticals.^13^ With this study, we showcased the effectiveness of the amine/nitrile pair as a potential LOHC (Figure 1).^10^

**Figure 1:**
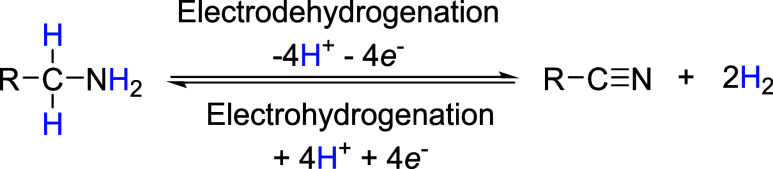
Electrodehydrogenation/electrohydrogenation of the amine/nitrile pair.

Recently, we have demonstrated the selective electrodehydrogenation of amines into their corresponding nitriles and molecular hydrogen using nickel-based electrodes (NiNF). In the same study, we employed thermocatalytic methods to facilitate the hydrogenation process (hydrogenation of amine), using molecular hydrogen gas under pressure as the reducing agent in aqueous media, along with a heterogeneous catalyst and elevated temperatures.^10^

To avoid thermocatalytic methods for the hydrogenation process, which require high temperatures,^14^ high pressures,^15^ and addition of additives,^16^ in the present study, we investigate the production of amines through electrocatalysis, which provides the benefit of operating under mild reaction conditions, utilizing water as solvent at room temperature and pressure, and employing more cost-effective catalysts.^3,17^

However, nitriles are challenging molecules to reduce due to the inert nature of the carbon–nitrogen triple bond. Their reduction requires high negative potential which impacts in faradaic fficiency (FE)^18^ of the transformation, probably due to a competition with Hydrogen Evolution Reaction (HER).

Moreover, the reduction of nitriles can lead to several side products, ranging from primary, secondary, and tertiary amines mixtures,^14,19,20^ to aldehydes.

Recently, several studies have successfully reduced nitriles to primary amines through electrochemical methods under highly basic or acidic conditions. For instance, some researchers have reported the electrohydrogenation of acetonitrile to ethylamine with high faradaic efficiency and selectivity using copper electrodes under anaerobic conditions.^13,21,22^ Moreover, Modestino and co-workers have reported the conversion of adiponitrile to hexamethylenediamine, a monomer for the production of nylon-6,6, using a Nickel Raney electrode applying a constant current density of −60 mA·cm^–2^.^23^ Zhang and co-workers have developed an electrochemical method for the reduction of benzylcyanide derivatives with pharmacological applications using Fe electrodes in an alkaline media,^24^ while Waldvogel et al. have reported a flow acidic electrolysis method for the continuous production of phenylethylamine from benzylcyanide, using Nickel Raney foam as electrodes.^25^

Accordingly, with the aim to enhance previously reported procedures, we describe a selective, mild, and efficient electrocatalytic method for the electrohydrogenation of nitriles into amines using an easy-to-prepare and inexpensive copper–silver electrode, under constant current density conditions, at ambient conditions, and in neutral pH media. While previous reports employed highly alkaline media, which can lead to the degradation of nitriles, we introduce, herein, a new approach that enables this transformation at neutral pH. This method not only demonstrates its potential for selective and efficient electroreduction but also moves closer to bridging laboratory-scale experiments with industrial applicability.

## Experimental Section

### Materials

All chemical reagents were obtained from commercial sources and used without further purification. All solutions were prepared in ultrapure water (Milli-Q gradient, ≥18.2 MΩ·cm). More information about the materials can be found on SI, Section S1.1.

### Electrodes Preparation

#### Copper Electrodes

CuE electrodes were prepared by electrodeposition of a Cu thin layer on top of the precleaned Cu foil in a two-electrode setup, as previously reported.^26^ Cu foils were cleaned via 30 min of sonication in H_2_O and ethanol, followed by drying with a N_2_ flow. Just prior to the electrodeposition, Cu foils were cleaned in a 10% HCl solution for 30 s to remove the native surface oxide layer, rinsed with Milli-Q type I ultrapure water, and dried in a N_2_ flow. The precleaned Cu foil was masked with a poly­(tetrafluoroethylene) tape to fix the exposed geometric area (1 cm^2^) as the working electrode (WE). Two Cu foils (4 cm^2^, each) were used as counter electrodes (CE) on both sides of the WE to ensure a homogeneous electrodeposition, in an undivided cell, as shown in Figure S1. An aqueous solution of 0.16 M CuSO_4_·5H_2_O in 0.6 M H_2_SO_4_ was used as the electroplating solution. Electrodeposition was carried out galvanostatically applying a constant current density of −60 mA·cm^–2^ for 10 min, keeping the distance (0.5 cm) between the cathode and anodes constant under magnetic stirring at 300 rpm. After the deposition, CuE electrodes were rinsed with H_2_O and dried under a N_2_ flow.

#### Copper–Silver Electrodes

CuEAg electrodes were prepared by a galvanic replacement technique. Previously prepared CuE electrodes were cleaned in a 10% HCl solution for 30 s, rinsed with H_2_O, and dried under a N_2_ flow, then CuE were dipped in a 10 mM AgNO_3_ solution for 1 min, until a black layer of Ag was deposited on top of the CuE. The CuEAg electrode was rinsed with H_2_O and dried under a N_2_ flow.

### Electrochemical Measurements

All of the electrochemical glassware was cleaned in HNO_3_ 10% solution overnight and then rinsed with copious amounts of Milli-Q type I ultrapure water. A Pt wire (99.95%, Thermo Scientific Chemicals), previously cleaned in HNO_3_ 70% solution and flame-dried, and Ag/AgCl electrode (3 M KCl, redoxme) were used as counter (CE) and reference electrodes (RE), respectively. Cyclic voltammetry (CV), electrochemical impedance spectroscopy (EIS), and chronopotentiometry (CP) experiments were performed with a three-electrode configuration, using a Gamry Reference 3000 potentiostat/galvanostat, and all solutions were purged with N_2_ for 20 min before each measurement.

All of the potentials are corrected versus reversible hydrogen electrode (RHE) according to eq 1, where *E*_RHE_ is the corrected potential *vs* RHE and *E*_Ag/AgCl_ applied potential *vs* Ag/AgCl (3 M KCl). The pH of the solutions was measured with a Crison ORP Sension+ PH3 pHmeter; for KCl and KHCO_3_, pH was 7.0 and 8.5, respectively,

1 $$E_{\mathrm{RHE}}=E_{\mathrm{Ag}/\mathrm{AgCl}}+0.197+0.059\cdot \mathrm{pH}$$

The electrochemical active surface area (ECSA) was obtained from measuring the frequency dependent impedance in a non-faradaic region of the system from 0 to −0.3 V, using EIS between 100 Hz and 100 kHz. The ECSA is calculated from the double-layer capacitance (*C*_dl_) according to eq 2, as previously reported by Jaramillo and co-workers,^27^ where *C*_s_ is the specific capacitance of a reference the sample.

2 $$\mathrm{ECSA}=\frac{C_{\mathrm{dl}}}{C_{s}}$$

#### Cyclic Voltammetry

The CV experiments were performed in a one-compartment cell in the absence and presence of 25 mM benzonitrile (BZN) in 10 mL of 0.5 M KCl as the electrolyte; all of the CV were measured at a scan rate of 5 mV·s^–1^ from −0.2 to −1.2 V *vs* RHE for 3 cycles, with CuE or CuEAg as WE.

#### Chronopotentiometry Experiments

The chronopotentiometric experiments (CP) were performed in a 3-electrode configuration with a commercial glass H-type cell (Ossila) divided by a previously activated Nafion 117 membrane (see Section S1.4), under magnetic stirring. The cathodic compartment contained 15 mL of the electrolyte with 25 mM of BZN while the anodic side contained the Pt as CE in 0.5 M Na_2_SO_3_. All of the CP experiments were run for 4 h, unless otherwise stated. Aliquots of 30 μL were taken from the working electrode compartment and diluted in 3 mL of water (LC-MS grade LiChrosolv, Sigma-Aldrich) for HLPC analysis.

Unless otherwise stated, all electrochemical experiments and product analyses were performed once under each condition. The optimal condition (CuEAg in 0.5 M KCl electrolyte, 70:30 H_2_O/CH_3_CN, *J* = −20 mA·cm^–2^, *t* = 4 h) was repeated three times to verify reproducibility, with the average value and standard deviation reported in the SI (see Tables S4–S7).

The conversion, yield, and faradaic efficiency (FE) were calculated according to eqs 3–5, respectively.

3 $$\%\;c\mathrm{onversion}=\frac{\mathrm{mol of consumed reactant}}{\mathrm{mol of initial reactant}}\cdot 100$$

4 $$\%\;y\mathrm{ield}=\frac{\mathrm{mol of specific product}}{\mathrm{mol of initial reactant}}\cdot 100$$

5 $$\%\;\mathrm{FE}=\frac{\mathrm{mol of}\;e^{-}\mathrm{consumed for specific product}}{\mathrm{mol of}\;e^{-}\mathrm{passed}}\cdot 100$$

#### HPLC Quantification

The separation and quantification of the products from the CP experiments were performed with a high-performance liquid chromatography (HPLC) Agilent 1260 Infinity II Quaternary System equipped with a 1260 DAD detector. The separation was performed using an InfinityLab Poreshell 120 EC-C18 column (4.6 mm × 100 mm, particle size 2.7 μm, Agilent) at 35 °C, an injection volume of 10 μL and isocratic elution of 30% acetonitrile (isocratic grade for liquid chromatography LiChrosolv, Sigma-Aldrich) and 70% 5 mM H_2_SO_4_ (99.9999% metals basis, 92% min, Thermo Scientific Chemicals) solution as mobile phase at a 0.5 mL/min flow rate and 0.1 mL/min^2^ flux gradient. The acid mobile phase was prepared in water (LC-MS grade LiChrosolv, Sigma-Aldrich). The elution time for each sample was 20 min. Compound identification and quantification were performed by using commercially available reagents. Calibration curves were done considering the relation between the maximum absorbance at different wavelengths and the concentration of the nitrile and amines compounds.

## Results and Discussion

### Copper and Copper–Silver Electrodes

Cu electrodeposited copper electrodes (CuE) were prepared by the electrodeposition of a thin layer of copper onto a commercially available delimitated area of the Cu foil (Figures 2 and S1). The electrodeposition process generates evolution of hydrogen gas from the water reduction reaction that influences the copper microstructure.^26^

**Figure 2:**
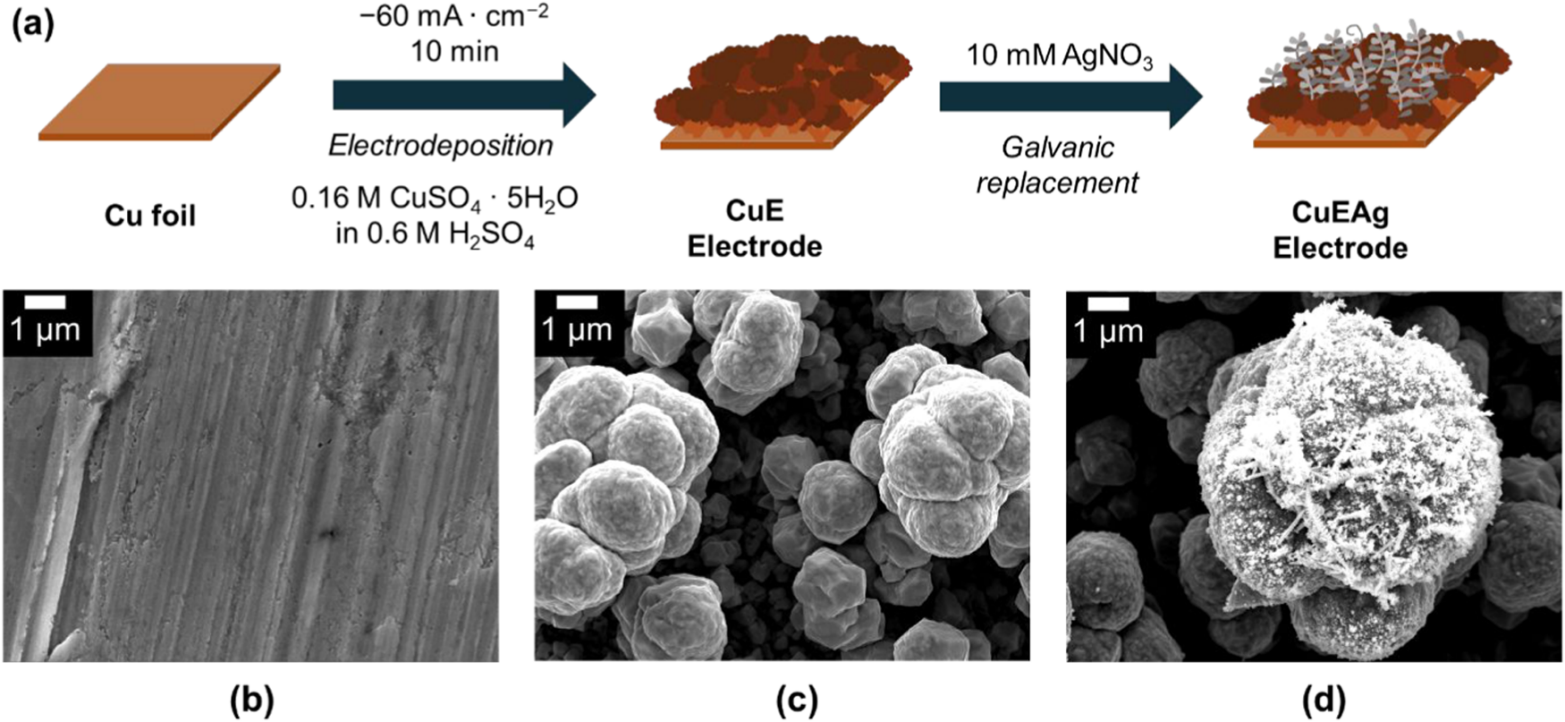
(a) Schematic illustration for the synthesis of CuE and CuEAg electrodes. (b–d) SEM images of the (b) Cu foil before electrodeposition of (c) CuE and (d) CuEAg electrodes.

During the electrodeposition procedure, the H_2_ bubbles operate as an *in situ* template preventing momentarily the contact between the copper solution and the copper cathode resulting in the formation of a crystalline Cu pore structure with good-adhesion to the Cu foil.^28^ From the scanning electron microscopy (SEM) images in Figure 2c, it can be noted how the Cu was electrodeposited onto the Cu foil forming a heterogeneous distribution of sizes and topologies in a cauliflower-like shape, hereafter denoted as CuE. Figure S2 shows the X-ray diffractogram (XRD) of CuE, revealing a face-centered cubic structure with high crystallinity and showing well-defined diffraction peaks at 2θ ≈ 43 and 50° corresponding to the (111) and (200) crystals planes.

The copper electrodeposited silver electrodes (CuEAg) were synthesized taking advantage of the potential difference between the Ag^0^/Ag^+^ and Cu^0^/Cu^2+^ redox pairs using the so-called galvanic replacement technique.^29^ This process involved immersing the CuE electrodes in a 10 mM AgNO_3_ solution. SEM analysis (Figure 2d) revealed the formation of Ag clusters on the surface of the CuE. The morphology of the Ag clusters corresponds to a dendritic structure, which is reported to enhance the electrode’s surface area while preserving electrical connectivity between the silver deposits.^29,30^ Characterization of the CuEAg electrodes by XRD only showed peaks corresponding to the Cu crystalline structure (Figure S2); however, energy-dispersive X-ray spectroscopy (SEM-EDS) confirmed the presence of silver clusters over the surface of the electrode (Figure S3).

### Electrochemical Hydrogenation of Benzonitrile

With the aim of studying the electrohydrogenation of nitriles into amines, we used the reduction of benzonitrile (BZN) to benzylamine (BZA) as a model reaction. BZA is a valuable building block for the chemical industry, besides the pair BZN/BZA can be used as a moderate LOHC with 4.0 wt % theoretical hydrogen storage capacity (HSC).

The electrohydrogenation of BZN to BZA requires the transfer of 4H^+^ and 4e^–^, (Figure 3a), with the initial hydrogenation step involving the addition of protons to form an imine intermediate (BI), which is then further hydrogenated to yield BZA. However, this electrochemical hydrogenation process is more complex, and a range of side products can be generated. For instance, over hydrogenation could lead to the formation of toluene (TOL).^24^ Additionally other possible side products include *N*-benzylidenebenzylamine (BIA) and dibenzylamine (DBA), formed through the coupling of imine and benzylamine, as well as benzamide and benzaldehyde, which result from the degradation of benzylamine and imine, respectively.^20^ A complete overview of the reaction pathway is provided in Figure S4. It is noteworthy that, in our study, we observed only trace amounts of these side products, with their distribution remaining below 5%, indicating that our Cu-based electrodes exhibit a high selectivity for the electrohydrogenation of nitriles to amines.

**Figure 3:**
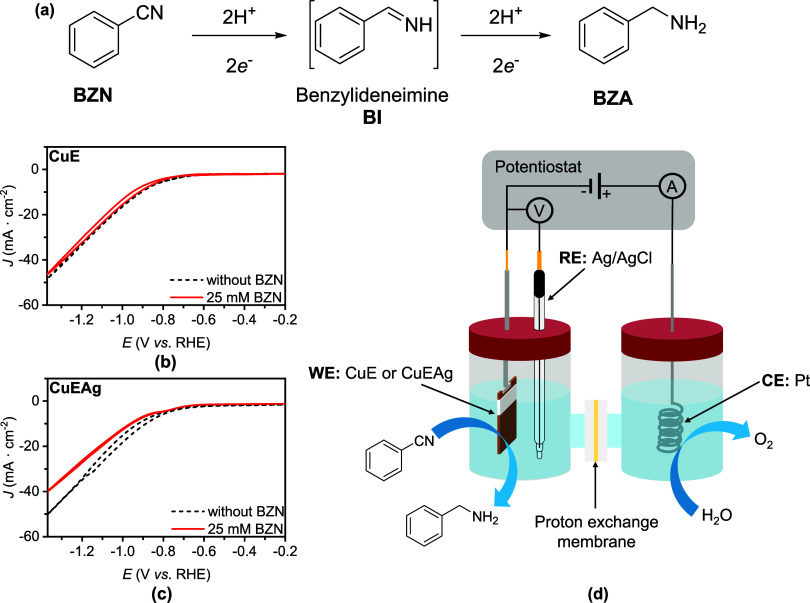
(a) Reaction pathway for electrohydrogenation of BZN. (b, c) Cyclic voltammetries of 25 mM BZN in 0.5 M KCl using (b) CuE and (c) CuEAg as electrodes, normalized by a geometrical area of 1 cm^2^. (d) Illustration of a two-compartment cell used for the electrohydrogenation of BZN to BZA.

The electrochemical properties of CuE and CuEAg electrodes were first analyzed by cyclic voltammetry (CV) with and without 25 mM BZN in 0.5 M KCl (pH = 7.0) as the electrolyte at a 5 mV·s^–1^ scan rate (Figure 3b,c). All of the potentials are correct *vs* RHE and the current density (*J*) is normalized by the geometrical area (1 cm^2^), unless otherwise stated. In the absence of BZN, the only current density observed is due to HER, with an onset at ca. −0.65 and −0.70 V for CuE and −0.60 to −0.70 V for CuEAg. The addition of BZN did not show the appearance of any significant reduction peak associated with the reduction of the organic molecule. The lack of a distinct reduction peak is most likely attributed to the concurrent competition between HER and the reduction of the organic molecule on CuE or CuEAg. Interestingly, the CuEAg electrode shows a minor decrease in *J* when BZN is present in the reaction media. This observation suggests an adsorption of the organic molecule on the surface of electrode, blocking actives sites for HER, as previously reported for this type of copper–silver electrodes.^29^ Additional evidence of substrate adsorption was obtained by varying the initial concentration of BZN. Cyclic voltammetry experiments were conducted under identical conditions but with higher concentrations of BZN (35 and 45 mM). The results revealed the absence of a distinct reduction peak in all cases (Figure S5). However, an important feature to highlight on the CV is the decrease of *J* as the concentration of BZN increases, which correlates with the adsorption of the organic molecule, hindering active sites for HER. This effect impacts the faradaic efficiency of the electrohydrogenation of BZN, as will be discussed later.

To further compare the CuE and CuEAg electrodes, we estimated the electrochemical surface area (ECSA) using electrochemical impedance spectroscopy (EIS) in the non-faradaic region, with a polycrystalline Cu foil as reference. Figure S6 shows the Nyquist plots obtained at −0.9 V, where no faradaic processes occur. The data were fitted using the simplified Randles circuit shown in the inset of Figure S6, following the methodology reported by Jaramillo and co-workers.^27^

The double-layer capacitance (*C*_dl_), extracted from EIS data, shows a marked increase for CuE and CuEAg compared to that for the Cu foil (see Figure S7), consistent with greater surface roughness introduced by electrodeposition and Ag incorporation. Furthermore, the estimated ECSA values, obtained from eq 2, indicate a progressive increase in surface area: ∼1.3 cm^2^ for Cu, ∼25 cm^2^ for CuE, and ∼108 cm^2^ for CuEAg. These results reveal an ∼25-fold enhancement in ECSA due to Cu electrodeposition and a further ∼4.3-fold increase upon Ag modification, highlighting the significant effect of surface roughening and Ag decoration in exposing active electrochemical sites, as noted from the SEM images (Figure 2b–d).

To investigate the best reaction conditions for the selective BZN electrohydrogenation, we performed chronopotentiometry experiments adjusting different conditions, such as cosolvent, applied current density (*J*), electrolyte, electrolyte concentration, and nitrile concentration, using CuE and CuEAg as working electrodes (WE), Ag/AgCl (3 M KCl) as the reference electrode (RE), and Pt wire as the counter electrode (CE). All of the chronopotentiometry experiments were performed in a two-compartment electrochemical cell, separated with a cation exchange membrane (Figure 3d) for 4 h, using 15 mL in each compartment and an exposed geometrical area of 1 cm^2^ for all WEs. From the chronopotentiometry experiments, the observed potentials range from −1.1 to −1.3 V *vs* RHE, depending on the working conditions. These results highlight the need for very negative potentials for nitrile electroreduction. All observed potentials (*E*_obs_) can be found on the SI, Tables S3–S8.

The detection, identification, and quantification of the organic substrates were done by high-performance liquid chromatography (HPLC), see Supporting Information for details (Tables S1–S2 and Figures S8–S10).

### Cosolvent Effect

Electrocatalytic transformations of organic molecules in water are limited by solubility issues. In order to favor solubility, a cosolvent is usually required. Due to the limited solubility of BZN in water, we assessed the influence of an organic cosolvent into the aqueous solution. We prepared solvent mixtures of 70:30 of H_2_O:X (v/v), where X = acetonitrile (CH_3_CN), 1,4-dioxane, and methanol (CH_3_OH), using 0.5 M KCl as the electrolyte. This mixture proportion has previously been reported for electroreduction of slightly soluble organic molecules in aqueous solutions.^31^ A higher concentration of the organic cosolvent induces the formation of two phases and/or the precipitation of KCl.

As observed in Figure 4 (see also Table S3 and Figure S11), the application of *J* = −30 mA·cm^–2^ using CuE as the electrode yielded the best results in terms of BZA yield (81%) and faradaic efficiency (FE) (23%) when acetonitrile was used as a cosolvent, compared to 1,4-dioxane and methanol. Moreover, with 1,4-dioxane, we observed an increase in the generation of side products (up to 11%), benzaldehyde (BAH) being the major, with a sharp decrease of FE (5%) for the production of BZA. In the case of using methanol as a cosolvent, lower conversion and yield values were obtained compared to acetonitrile, in addition to a significant difference between yield and conversion indicating a lower selectivity.

**Figure 4:**
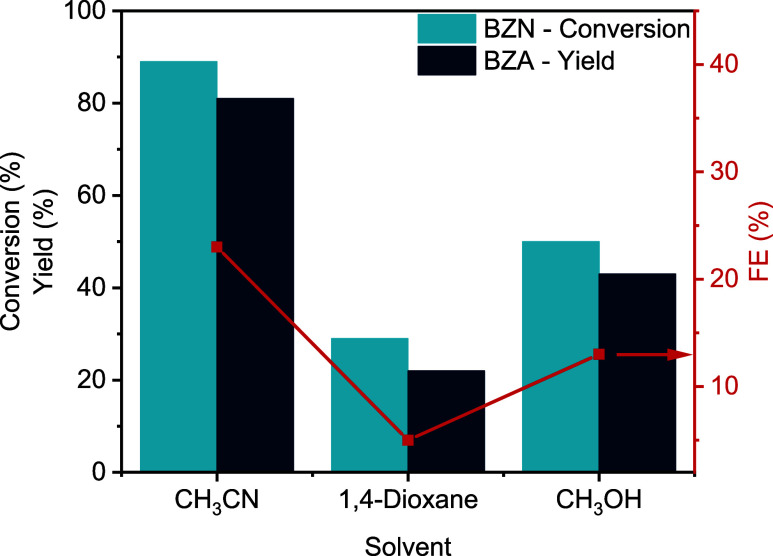
Effect of the cosolvent on the electrohydrogenation of BZN using CuE as the electrode and 0.5 M KCl in a 70:30 H_2_O:X mixture (X = CH_3_CN, 1,4-dioxane, CH_3_OH) applying a −30 mA·cm^–2^ constant current density for 4 h.

The use of a cosolvent improves nitrile solubility in water and enhances mass transfer rates of organic molecules to the electrode.^23^ Given the best results using a H_2_O:CH_3_CN mixture, our next studies in the electrohydrogenation of nitriles were performed with such solvent combination.

### Current Density Effect

The effect of current density on the electrohydrogenation of BZN was evaluated with three different *J* (−10, −20, and −30 mA·cm^–2^) using CuE and CuEAg with 0.5 M KCl as the electrolyte (Table S4 and Figure S12).

As depicted in Figure 5, CuE demonstrates moderate conversion (58%) and yield (56%) at −10 mA·cm^–2^, with FE equal to 40%. In contrast, CuEAg shows a significantly lower conversion (26%) and yield (22%) under the same conditions, with a FE of 24%. However, as the current density increases to −20 and −30 mA·cm^–2^, the CuEAg electrode’s performance improves markedly, surpassing that of the Cu electrode at these higher current densities. This behavior suggests that the CuAg surface requires higher overpotentials to facilitate effective hydrogen adsorption and subsequent nitrile reduction.

**Figure 5:**
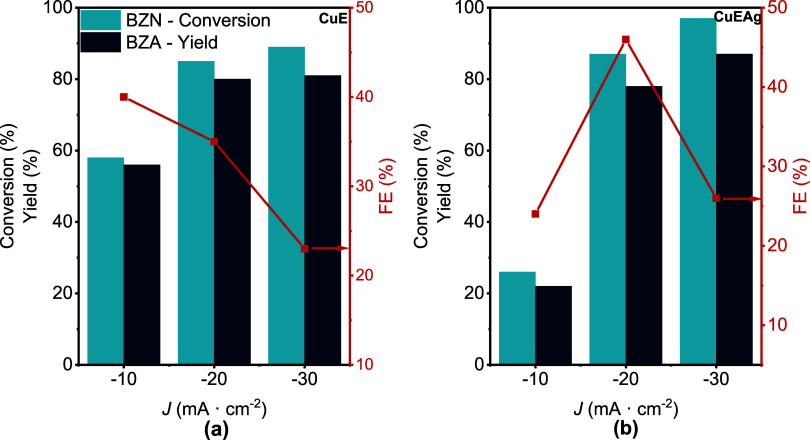
Effect of current density on electrohydrogenation of 25 mM BZN in 0.5 M KCl 70:30 H_2_O:CH_3_CN solution and 4 h reaction using (a) CuE and (b) CuEAg as electrodes. The legend in panel (a) also applies for panel (b).

The distinct electrochemical behaviors between CuE and CuEAg electrodes can be attributed to their differing hydrogen adsorption properties. Previous reports have shown that Ag-modified Cu surfaces exhibit altered hydrogen adsorption characteristics, which can influence catalytic activity, thereby impacting their catalytic performance in HER.^32–34^

Therefore, the enhanced performance of the CuAg electrode at higher current densities may result from the increased availability of active hydrogen species required for the electrohydrogenation of BZN. This observation highlights the importance of optimizing *J* based on the specific electrode material to achieve efficient catalytic conversion. However, the use of higher current densities may also lead to a competition increase between HER and the nitrile reduction.

A key feature of our system is its high selectivity toward BZA. Only trace amounts (yields <5%) of side products, such as benzamide (BZM), benzoic acid (BZ), and BAH, were detected by HPLC (Figure S12). Consequently, the formation of side products does not pose a limitation in the conversion of nitriles to amines using CuE and CuEAg electrodes.

It is also worth noticing that the introduction of Ag in the Cu electrodes helps improve the faradaic efficiency, from 35% (CuE) to 46% (CuEAg) (Figure 5) at −20 mA·cm^–2^, suggesting a synergy effect between Cu and Ag. The synergistic effect between Cu and Ag has previously been reported in the electrocatalytic reduction of 5-hydroxymethylfurfural.^29^ In this regard, Cu can play an important role with the adsorption of the organic molecules over the electrode surface, while Ag impedes HER, providing an improvement in faradaic efficiency. Interestingly, no reaction conversion was observed when a Ag foil was used as the electrode alone (Figure S13), reinforcing our statement about the critical role of decorating the Cu electrode surface with Ag.

All of these observations suggest that −20 mA·cm^–2^ shows the better results in terms of conversion, yield, and FE with both electrodes. Following experiments were performed using that current density.

Moreover, to confirm the stability of acetonitrile (*i*.*e*. CH_3_CN electroreduction) under the applied electrochemical conditions, control experiments were performed using the same H_2_O/CH_3_CN mixture in the absence of benzonitrile, applying −20 m·cm^–2^ for 4 h using both electrodes (CuE and CuEAg). Post-electrolysis analysis by NMR showed no evidence of acetonitrile reduction products such as ethylamine (see Figures S14 and S15), suggesting that CH_3_CN remains stable under our working conditions.

### Electrolyte Effect

With the aim to improve the faradaic efficiency of the process, the effect of the electrolyte on the electrohydrogenation using potassium chloride (KCl) and potassium bicarbonate (KHCO_3_) in a 0.5 M concentration was studied. Previous reports on acetonitrile electrohydrogenation employed bicarbonate as the electrolyte, obtaining successful inhibition of HER.^21^ Strong basic media, such as KOH (pH = 14), were not tested to avoid the known degradation of BZN to side products, such as BZM and BZ (Figure S4).^35^

In our case, the use of KHCO_3_ did not help to improve FE with any electrode, attaining FE of ∼22% with CuE or ∼34% for CuEAg as noted in Figure 6 (see also Table S5 and Figure S16). Although KHCO_3_ was initially evaluated as an electrolyte, the system showed a poor performance and was not pursued further. It is noted that KHCO_3_ can act as a CO_2_ reservoir, potentially enabling CO_2_ reduction on Cu electrodes and that local pH shifts because bicarbonate equilibrium could influence reaction selectivity.^36–39^ To avoid these complications and ensure a clearer interpretation of electroreduction pathways, subsequent experiments were conducted in the unbuffered KCl electrolyte, achieving better BZN conversion and BZA yield with both electrodes.

**Figure 6:**
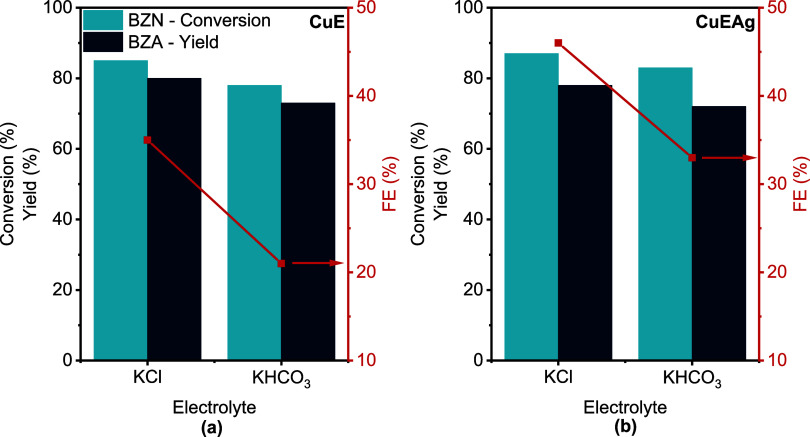
Effect of the electrolyte on the electrohydrogenation of 25 mM BZN in 0.5 M X (X = KCl, KHCO_3_) 70:30 H_2_O:CH_3_CN solution using (a) CuE and (b) CuEAg as electrodes at −20 mA·cm^–2^ and 4 h reaction. The legend in panel (a) also applies for panel (b).

Several authors shared insights on how Cl^–^ anions can interact with the electrode surface, altering or partially blocking electroactive sites, which can influence, by facilitating or suppressing, specific reactions pathways.^40–42^ To further support this claim, we performed electrochemical *in situ* surface-enhanced Raman spectroscopy (SERS) to elucidate the Cl^–^ ion adsorption on the electrodes surface. The Raman spectra (see Figure S17) show the emergence of low-frequency bands around 250–280 cm^–1^, assignable to Cu–Cl and Ag–Cl stretching vibrations, consistent with chloride adsorption on the electrode surface.^43–46^ These features increase with more negative potentials, supporting the role of Cl^–^ in modifying the surface and possibly suppressing HER.

Furthermore, the band observed in the 2200–2300 cm^–1^ region corresponds to ν­(C≡N) vibrations. While this mode is characteristic of benzonitrile (∼2225 cm^–1^), acetonitrile also exhibits a strong C≡N stretch in this range (∼2253 cm^–1^), and therefore both may contribute in the spectra.^22,24^

### Electrolyte Concentration Effect

Subsequently, the analysis of the effect of KCl concentration in the electrohydrogenation of BZN was evaluated with 0.1, 0.5, and 1.0 M of KCl, applying a constant current density of −20 mA·cm^–2^ with CuE and CuEAg as electrodes (Table S6 and Figure S18).

Figure 7 shows similar conversion, yield, and FE with CuE and CuEAg as electrodes, when a 0.1 or 0.5 M KCl solution was used. However, at 1.0 M KCl, a considerable drop in FE was observed, suggesting that high concentrations of KCl could favor competing reactions or other processes on the electrode surface. Apparently, Cl^–^ ions could compete more effectively with BZN for adsorption sites, reducing the efficiency of electrohydrogenation to BZA. As previously noted, CuEAg electrodes once again demonstrate superior FE across the entire electrolyte concentration range.

**Figure 7:**
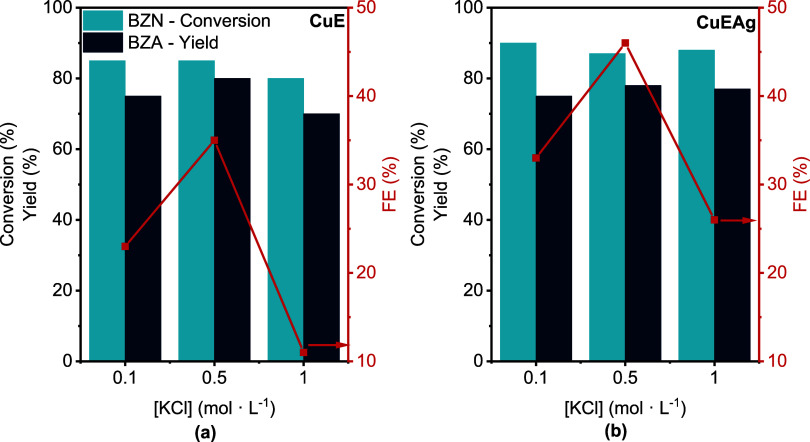
Effect of the electrolyte concentration on the electrohydrogenation of 25 mM BZN in 0.1, 0.5, and 1.0 M KCl 70:30 H_2_O/CH_3_CN solution using (a) CuE and (b) CuEAg as electrodes at −20 mA·cm^–2^ and 4 h reaction. The legend in panel (a) also applies for panel (b).

Although the experiments presented in Figure 7 were carried out galvanostatically under identical conditions (current density, time, and initial BZN concentration), FEs show a more pronounced variation than the corresponding yields. This divergence could be related to electrolyte-dependent changes in reaction selectivity. At higher salt concentrations, the increased ionic strength can influence interfacial properties, including the structure of the electric double layer, ion mobility, and local pH gradients, which, in turn, affects the competition between BZN electroreduction and side reactions such as HER.^40,47–49^ Thus, even when similar product quantities are generated (*i*.*e*., similar yields), the efficiency with which charge is converted to product decreases as a larger portion of the current is consumed by HER, resulting in a reduced FE. These results show the importance of electrolyte composition in controlling selectivity and efficiency in electrochemical systems.

### Benzonitrile Concentration Effect

Next, we tested the effect of BZN concentration using CuEAg as the electrode. By applying a constant *J* = −20 mA·cm^–2^, with a 45 mM BZN solution, we were able to improve FE up to 63%, as shown in Figure 8a (Table S7 and Figure S19). This behavior can be explained by the increased availability of reactant molecules near the electrode surface at higher nitrile concentrations, which enhances the probability that the electrons transferred to the electrode are utilized for electrohydrogenation. Conversely, at lower BZN concentrations (15 mM), the limited number of reactant molecules near the electrode surface results in a lower FE. For example, using a highly diluted BZN solution (15 mM), a FE as low as 19% was achieved for a 4 h reaction (Figure 8a).

**Figure 8:**
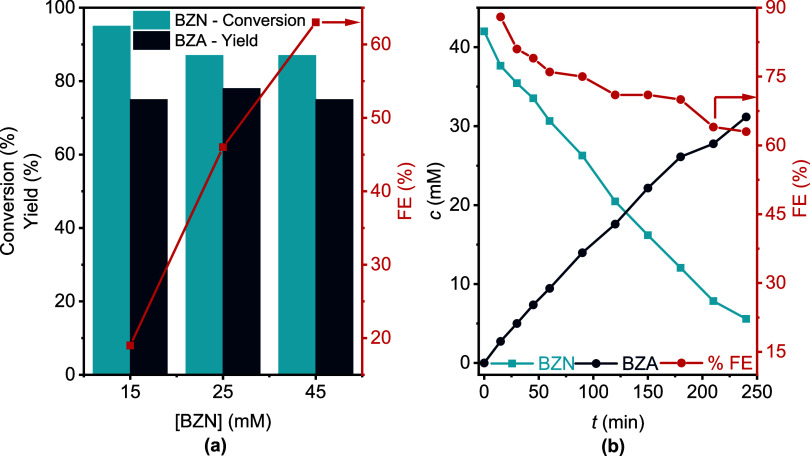
(a) Effect of the BZN concentration on the electrohydrogenation reaction in 0.5 M KCl 70:30 H_2_O/CH_3_CN electrolyte solution using CuEAg as the electrode at −20 mA·cm^–2^ and 4 h reaction. (b) Kinetic experiment for 4 h of a 45 mM BZN electroreduction reaction to BZA, using CuEAg as the electrode and an applied *J* = −20 mA·cm^–2^.

Reaction monitoring profiles of concentration *vs* time unveiled an increasing conversion of the substrate into the final product. We have not observed the presence of side products in significant amounts (Figures 8b and S20). Furthermore, to confirm the absence of over-reduction of the amine, we conducted an experiment under our working conditions using 25 mM BZA in 0.5 M KCl to determine if toluene (TOL) could be produced. However, no conversion of benzylamine was observed, indicating the high selectivity of CuEAg in the reduction of nitriles (Figure S21).

Reaction monitoring profiles indicate a high FE at shorter reaction times, reaching up to 80% at 30 min (Figure 8b), followed by a decline as the reaction progresses. This decrease of FE with reaction time might be associated with a competition between HER and nitrile electrohydrogenation. To further verify competition with HER, we decided to connect the electrochemical cell to a micro-GC. The results confirmed the presence of a H_2_ peak during the curse of the reaction indicating that, effectively, the low FE observed is caused by competition with HER (Figure S22).

### Deuterated Experiments

Mechanistic studies were performed to understand the electrohydrogenation of nitriles using deuterated solvents. Initially, we conducted experiments in deuterated water (D_2_O) to confirm that the source of protons in the final benzylamine came from the water molecule. In this case, we did an experiment using 25 mM 4-methoxybenzonitrile in 0.5 M KCl 70:30 D_2_O/CH_3_CN, using CuEAg as the electrode and applying a constant *J* = −20 mA·cm^–2^ for 6 h. From the ^1^H NMR spectra (Figure S23), we can see the disappearance of the deuterated methylene group (CD_2_) due to the formation of 4-methoxybenzylamine. Moreover, from ^2^H NMR (Figure S24), we can detect a singlet associated with the CD_2_ moiety from 4-methoxybenzylamine. At the ^13^C NMR, we can see a multiplet due to C–D coupling (*J*_(C–D)_ = 22 Hz) (Figure S25). As is well-known the coupling constant for ^13^C–^2^H (*J*_(C–D)_) is typically around 20–25 Hz, which is distinct from the ^13^C–^1^H coupling constant (*J*_(C–H)_, around 125–135 Hz).^50^ This lower value obtained from *J*_(C–D)_ reflects the weaker interaction between ^13^C and ^2^H compared to ^13^C and ^1^H. Deuteration experiments verified that water acts as the proton source in the reduction of nitriles.

### Reaction Scope

We investigated the efficiency and versatility of our CuEAg electrode for the electrohydrogenation of different aromatic nitriles using the optimal reaction conditions previously detailed. Due to the low solubility of the more substituted aromatic nitriles, we worked with a 25 mM concentration of the corresponding nitrile in a 0.5 M KCl 70:30 H_2_O:CH_3_CN solvent mixture. All of the products were detected and quantified by HPLC through calibration curves of each nitrile/amine pair (Tables S2 and Figure S10).

Figure 9 shows all of the results of the electrohydrogenation of different aromatic nitriles. Conversion, yield, and FE obtained are presented in Table S8 (Figure S26). Aromatic nitriles **2a** and **3a** yielded low quantities of the corresponding amines (29 and 50% for **2b** and **3b**, respectively), while a substitution on the *para* position on **4a** gave a better yield (67%) and FE (42%) of **4b**. These results could be related to the adsorption of the nitrile and steric hindrance on the *ortho* and *meta* positions, delaying the hydrogenation step.

**Figure 9:**
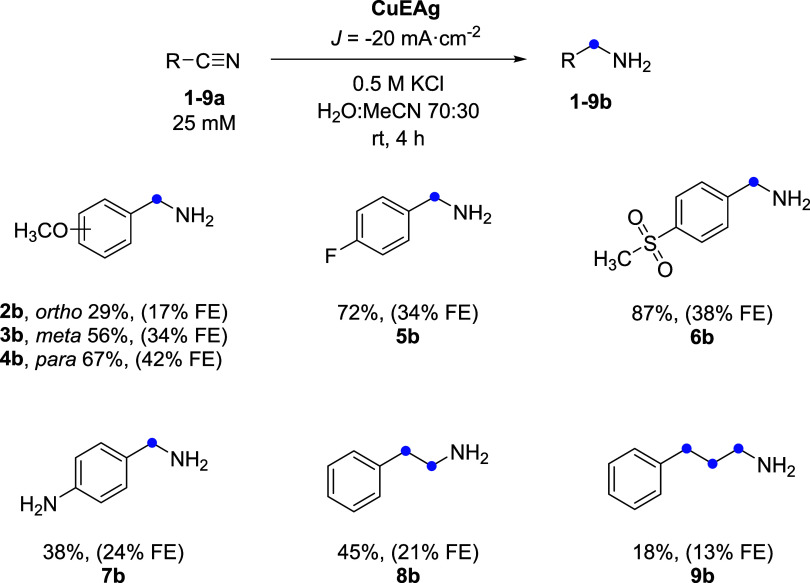
Synthetic scope for the electrohydrogenation of aromatic nitriles. Reaction conditions: two-compartment H-cell with 25 mM aromatic nitrile in 0.5 M KCl 70:30 H_2_O/CH_3_CN in the cathode and 0.5 M Na_2_SO_3_ in the anode compartment. Reactions were performed in a three-electrode configuration with CuEAg as WE, Ag/AgCl (3 M KCl) as RE, and Pt as CE, applying *J* = −20 mA·cm^–2^ and a 4 h reaction. For each amine, the yield and FE (in parentheses) were calculated from HPLC quantifications.

The presence of a second and third CH_2_ moiety in the nitrile structure (**8a**–**9a**) did not enhance the yield of the corresponding amines (**8b**–**9b**). This decrease in yield is likely attributed to the reduced solubility of these compounds and the disruption of electronic delocalization between the aromatic ring and the nitrile group compared to BZN.

### Green Chemistry Aspects

Electrohydrogenation presents significant advantages for the conversion of nitriles to amines, aligning closely with the principles of green chemistry, by offering a more environmentally friendly alternative compared to thermal catalysis, since it can operate directly on aqueous conditions, using water as the proton source, operating under ambient temperature and pressure, and using electricity as the driving force, ideally sourced from renewables.^51,52^

We evaluated several green chemistry metrics, including economic aspects (Eco), atom economy (AE), effective mass yield (EMY), and safety considerations (see SI Section S11).^25,53^ Our method utilizes water as both the solvent and proton source, eliminating the need for external hydrogen gas and the associated risks of high temperatures and pressures. Furthermore, the use of a neutral pH avoids the degradation of nitriles in highly alkaline media, helps to enlarge the lifetime of electrodes, and allows the use of a wide variety of materials in the construction of the reactors, which are key aspects for the scale-up and industrial exploitation of the reaction.

While our method achieved a moderate atom efficiency due to the oxygen evolution reaction at the anode, the resulting oxygen can be considered a benign byproduct. Overall, the electrohydrogenation process minimizes hazardous materials, reduces wasteful side products, and can be powered using renewable energy sources, underscoring its potential as a sustainable and safer alternative for nitrile hydrogenation, compared to thermal hydrogenation, which suffers from poor selectivity due to over hydrogenation or side reactions.^54^

Standard green chemistry metrics do not account for the FE. This parameter indicates how much of the total current (and thus power) in the reaction is used to produce the desired products. Including the FE in sustainability assessments is essential to complete the green chemistry metrics. It is worth noting that a low FE does not necessarily imply that a reaction is unsustainable. If side products have practical uses or value, they can contribute to the overall sustainability of the process.

In our experiments, we observe a high FE (>80%) at the beginning of the reaction (Figure 8b) when the concentration of BZN is high (>35 mM). As BZN is consumed, the FE gradually decreases, producing H_2_, which is a valuable byproduct. A high FE can be maintained in flow systems with continuous feed, though this setup requires further investigation.

Despite the low solubility of BZN in water and the requirement for relatively high overpotentials, this method circumvents the need for high-pressure hydrogen gas, improves safety, and allows for precise control over reaction conditions. Moreover, electrochemical methods also enable localized reaction environments (*e*.*g*., near-electrode pH and proton flux) that can be exploited for selectivity, especially when using tailored electrodes such as Cu–Ag composites. As shown in our work, reasonable yields and FE are achievable under mild ambient conditions, suggesting that further optimization of interfacial properties and reactor design could address current mass transfer limitations.

## Conclusions

In this study, we have demonstrated the successful electrohydrogenation of benzonitrile into benzylamine using copper and copper–silver electrodes (CuE and CuEAg, respectively) at neutral pH. We have proven that the introduction of Ag into the Cu structure (CuEAg electrode) significantly enhanced the Faradaic efficiency (FE) at larger current densities. This superior performance of CuEAg is attributed to the synergies between Cu and Ag, where Cu facilitates the adsorption of organic molecules while Ag delays the onset of HER, thereby enhancing the overall FE.

Interestingly, at lower current density (−10 mA·cm^–2^), the CuE electrode outperforms CuEAg in terms of yield and FE. However, as the current density increases to −20 and −30 mA·cm^–2^, CuEAg consistently exhibits superior FE, even though the conversion and yield are comparable to those of CuE. This behavior suggests that the Cu–Ag synergy is more pronounced at higher reaction rates, where the Ag component likely plays a role in suppressing the competing HER.

NMR experiments in deuterated water (D_2_O) confirmed that water acts as the proton source for the hydrogenation of nitriles, highlighting the advantage of electrochemical methods over conventional reduction techniques, which often require high temperatures and hazardous reagents. Thus, our approach provides a safer and more environmentally friendly alternative for nitrile reduction with a high selectivity toward the amine. Overall, this study demonstrates the potential of Cu-based electrodes in electrochemical organic transformations at neutral pH, with promising prospects for green chemistry applications.

## Supplementary Material



## Notes

Experimental section, materials details, characterization of CuE and CuEAg electrodes, benzonitrile hydrogenation reaction pathways, cyclic voltammetry experiments at different BZN concentrations, electrochemical active surface area of CuE and CuEAg electrodes, product separation and quantification with HPLC, benzonitrile electroreduction reaction optimization, detection of H_2_ from the electrohydrogenation of benzonitrile, NMR deuteration experiments, electrohydrogenation reaction scope results and green chemistry metrics (PDF)
