# Supplementary material for: Electrohydrogenation of Benzonitrile into Benzylamine under Mild Aqueous Conditions
Source: ACS Sustain Chem Eng. 2025 Jun 5;13(23):8660–70. doi: 10.1021/acssuschemeng.5c02168 (PMC12175222; doi:10.1021/acssuschemeng.5c02168)
Supplement: Supplementary file 1 [file sc5c02168_si_001.pdf]

SUPPORTING INFORMATION

Electrohydrogenation of benzonitrile into benzylamine under mild aqueous conditions

Jose Solera-Rojas,[1] Carles Forés,[1] Guillem Beltrán-Gargallo,[1] Francisco Fabregat-Santiago,[1] José A. Mata[1], Carmen Mejuto[1*] and Elena Mas-Marzá[1*]

[1]Institute of Advanced Materials (INAM), Universitat Jaume I, 12006 Castelló, Spain.
*E-mail: mejuto@uji.es, emas@uji.es

This file includes:

Number of Pages: 37

Number of Figure: 25

Number of Tables: 10

Table of Contents

S1. Experimental Section ***S3***

S1.1. Materials **S3**

S1.2. Copper electrodes preparation **S4**

S1.3. Chronopotentiometry experiments. **S4**

S1.4. Nafion™ 117 membrane activation. **S4**

S1.5. Characterization Techniques. **S4**

S1.5.1. Scanning Electron Microscopy. S4

S1.5.2. X-Ray Diffraction Pattern. S5

S1.5.3. Nuclear Magnetic Resonance Spectroscopy. S5

S1.5.4. Micro Gas Chromatography. S5

S1.5.5. Electrochemical *in situ* surface-enhanced Raman spectroscopy (SERS). S5

S2. Characterization of CuE and CuEAg electrodes. ***S6***

S3. Benzonitrile hydrogenation reaction pathways. ***S8***

S4. Cyclic voltammetry experiments at different BZN concentrations. ***S9***

S5. Electrochemical active surface area of CuE and CuEAg electrodes. ____ *S9*

S6. Product Separation and Quantification with HPLC. ____ *S11*

S7. Benzonitrile Electroreduction Reaction Optimization. ____ *S18*

S8. Detection of H_2 from the electrohydrogenation of benzonitrile. ____ *S28*

S9. NMR deuteration experiments. ____ *S29*

S10. Electrohydrogenation Reaction Scope Results. ____ *S32*

S11. Green Chemistry Metrics. ____ *S35*

S12. References ____ *S38*

S1. Experimental Section

S1.1. Materials

The materials included are Cu foil (99.9%, 0.2 mm thickness, GoodFellow, Inc.), Ag foil (99.95%, 0.5 mm thickness, GoodFellow, Inc.), $CuSO_4 \cdot 5H_2O$ (99.999% trace metal basis, Sigma-Aldrich), H_2SO_4 (ACS reagent, 95.0-98.0%, Sigma-Aldrich), HCl (ACS reagent, 37%, Sigma-Aldrich), HNO_3 (ACS reagent, 70%, Sigma-Aldrich), $AgNO_3$ (≥99.0%, ACS Reagent, Sigma-Aldrich), H_2O_2 solution (30% w/w, puriss, Sigma-Aldrich), ethanol (96% vol, VWR Chemicals), CH_3CN (isocratic grade for liquid chromatography LiChrosolv®, Sigma-Aldrich), CH_3CN (99.9+% extra dry, Thermo Scientific), KCl (99.999% trace metal basis, Thermo Scientific), 1,4-dioxane (99.8%, anhydrous, Sigma-Aldrich), CH_3OH (≥99.8%, ACS Reagent, Sigma-Aldrich), $KHCO_3$ (≥99.95% trace metals basis, Sigma-Aldrich), Na_2SO_3 (98%, anhydrous, Thermo Scientific), benzonitrile (99%, ReagentPlus®, Sigma-Aldrich), benzylamine (99%, ReagentPlus®, Sigma-Aldrich), (*E*)-*N*-Benzylidene-1-phenylmethanamine (95+%, Fluorochem), benzaldehyde (≥99%, Sigma-Aldrich), benzamide (99%, Sigma-Aldrich), benzoic acid (99%, ReagentPlus®, Sigma-Aldrich), dibenzylamine (97%, Sigma-Aldrich), toluene (99.8%, anhydrous, Sigma-Aldrich), *N*-benzylethanamine (97.0% Fluorochem), 2-methoxybenzonitrile (99%, Sigma-Aldrich), 2-methoxybenzylamine (98%, Sigma-Aldrich), 3-methoxybenzonitrile (98%, Sigma-Aldrich), 3-methoxybenzylamine (98%, Sigma-Aldrich), 4-methoxybenzonitrile (99%, Sigma-Aldrich), 4-methoxybenzylamine (98%, Sigma-Aldrich), 4-fluorobenzonitrile (99.0%, Fluorochem), 4-fluorobenzylamine (97.0%, Fluorochem), 4-(methylsulfonyl)benzonitrile (95.0%, Fluorochem), 4-(methylsulfonyl)benzylamine (95.0%, Fluorochem), benzylcyanide (98%, Sigma-Aldrich), phenethylamine (≥99%, Sigma-Aldrich), 3-phenylpropionitrile (99%, Sigma-Aldrich), 3-phenylpropylamine (98.0%, Fluorochem), 4-aminobenzonitrile (98.0%, Fluorochem), 4-(aminomethyl)aniline (95.0%, Fluorochem), D_2O (99.97% D, Eurisotop) and ethylamine solution (66.0-72.0% in H_2O, Sigma-Aldrich).

S1.2. Copper electrodes preparation

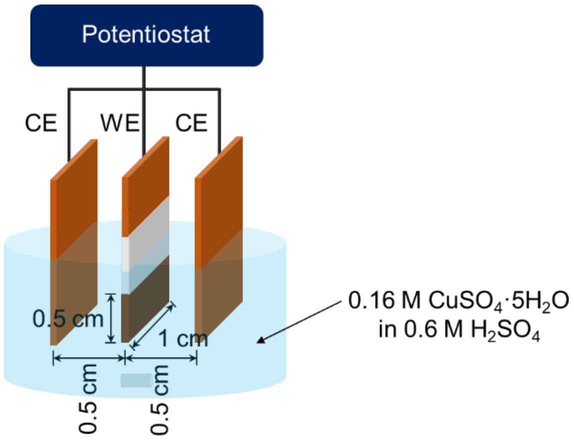

Figure S1. Schematic illustration of copper electrodeposition on top of Cu foil.

S1.3. Chronopotentiometry experiments.

For the chronopotentiometry experiments (CP) an exposed geometrical area of 1 cm^2 was used in with all the WEs, different conditions were tested using 15 mL of a 25 mM BZN solution in the cathodic compartment: (a) varying the organic solvent composition of a 70:30 H_2O:X electrolyte mixture, where X = CH_3CN, 1,4-dioxane and CH_3OH with 0.5 M KCl each, applying a J = -30 mA·cm^{-2} and CuE as WE; (b) applying different current densities (-10, -20 and -30 mA·cm^{-2}) in a 0.5 M KCl solution in a 70:30 H_2O:CH_3CN solution, using CuE and CuEAg as WE; (c) varying the type of electrolyte (KCl and $KHCO_3$) maintaining a concentration of 0.5 M in a 70:30 H_2O:CH_3CN solvent mixture, using CuE and CuEAg as WE and applying a J = -20 mA·cm^{-2}; (d) varying BZN concentration (15, 25, 45 mM) in a 0.5 M KCl 70:30 H_2O:CH_3CN solvent mixture, with CuEAg as WE and applying a J = -20 mA·cm^{-2}.

S1.4. Nafion™ 117 membrane activation.

The Nafion™ 117 membrane (0.180 mm thick, ≥0.90 meq/g exchange capacity, Sigma-Aldrich) was activated by heating at 80 ºC for 1 h each in a 3% H_2O_2 solution, H_2O, 2 M H_2SO_4 solution and H_2O. The activated membranes were stored in H_2O.

S1.5. Characterization Techniques.

S1.5.1. Scanning Electron Microscopy. The surface morphology of the electrodes was examined by Scanning Electron Microscopy – Energy dispersive X-ray

spectroscopy (SEM-EDX) on a JEOL 7001F microscope operating at 15 kV equipped with a Leica Zeiss LEO 440.

S1.5.2. X-Ray Diffraction Pattern. X-ray diffraction pattern (XRD) were measured in a D8 Advance Brucker-AXS diffractometer; wide-angle diffractogram using grazing incidence diffraction over 2θ range from 10 to 70º with a step size of 0.050º and counting time per step 3 s.

S1.5.3. Nuclear Magnetic Resonance Spectroscopy. ^{1}H and ^{13}C-NMR were collected on a 400 MHz Bruker spectrometer and ^{2}H-NMR were measured in a 500 MHz Oxford spectrometer. For the NMR deuteration experiment, a CP was performed using a 25 mM solution of **3a** in 0.5 M KCl D_2O:CH_3CN 70:30 solvent mixture, using CuEAg as electrode for 6 h, applying -20 $mA{\cdot}cm^{-2}$ in a two-compartment cell, separated by a Nafion™ 117 membrane previously activated and stored in D_2O. From the experiments, aliquots of 500 μL were taken at the beginning and end of the reaction and directly taken to the NMR.

S1.5.4. Micro Gas Chromatography. The detection of H_2 was performed using a Micro GC 490 Agilent Technologies coupled with a closed electrochemical H-cell.

S1.5.5. Electrochemical *in situ* surface-enhanced Raman spectroscopy (SERS). SERS experiments were performed with a Raman WITec alpha300 apyron, a 532 nm laser was employed. A Zeiss 20X objective was used for *in situ* experiments, conducted in a commercial 3-electrode *in situ* Raman cell from redox.me, connected to an Autolab PGSTAT204 Potentiostat/Galvanostat. Typical spectra acquisition times were 20-30 s. Spectra were first acquired at the most positive potential and was progressively increased in a chronoamperometric scan of 2 min, each step. Cosmic ray removal was applied during the spectroscopic experiment.

S2. Characterization of CuE and CuEAg electrodes.

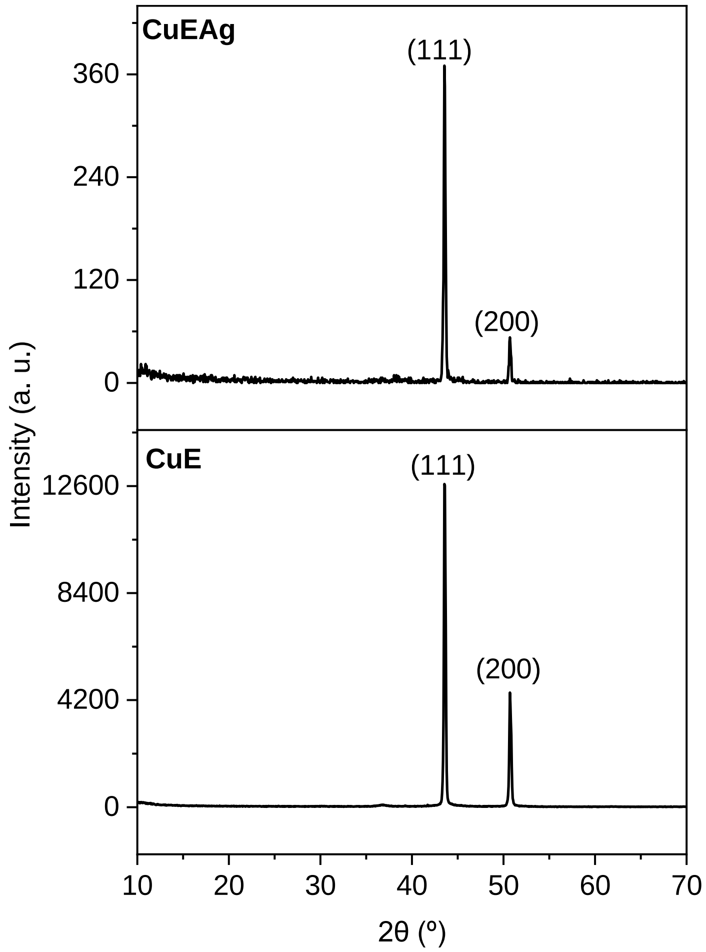

Figure S2. XRD pattern of CuE and CuEAg electrodes showing well-defined diffraction peaks at $2\theta \approx 43^{\circ}$ and 50° corresponding to the (111) and (200) planes of Cu, respectively, in a face-centered cubic lattice.

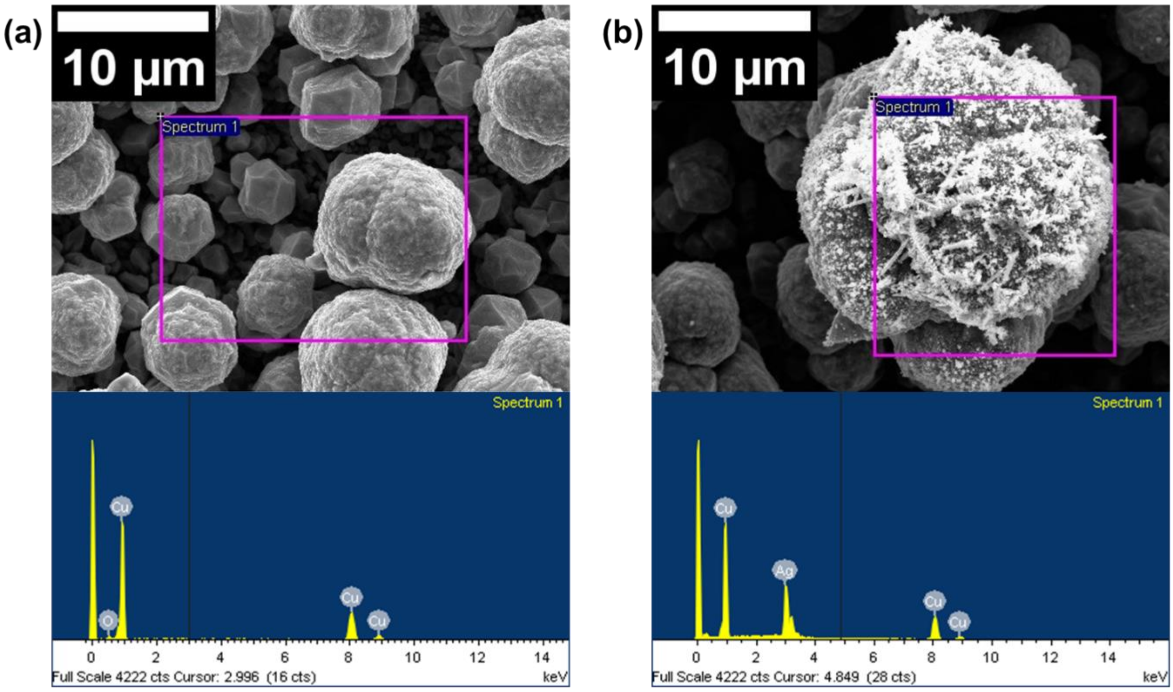

Element	Weight %	Atomic %
O K	1.39	5.29
Cu K	98.61	94.71
Totals	100.00	

Element	Weight %	Atomic %
Cu K	62.80	74.13
Ag l	37.20	25.87
Totals	100.00	

Figure S3. SEM-EDS analysis of **(a)** CuE and **(b)** CuEAg electrodes.

S3. Benzonitrile hydrogenation reaction pathways.

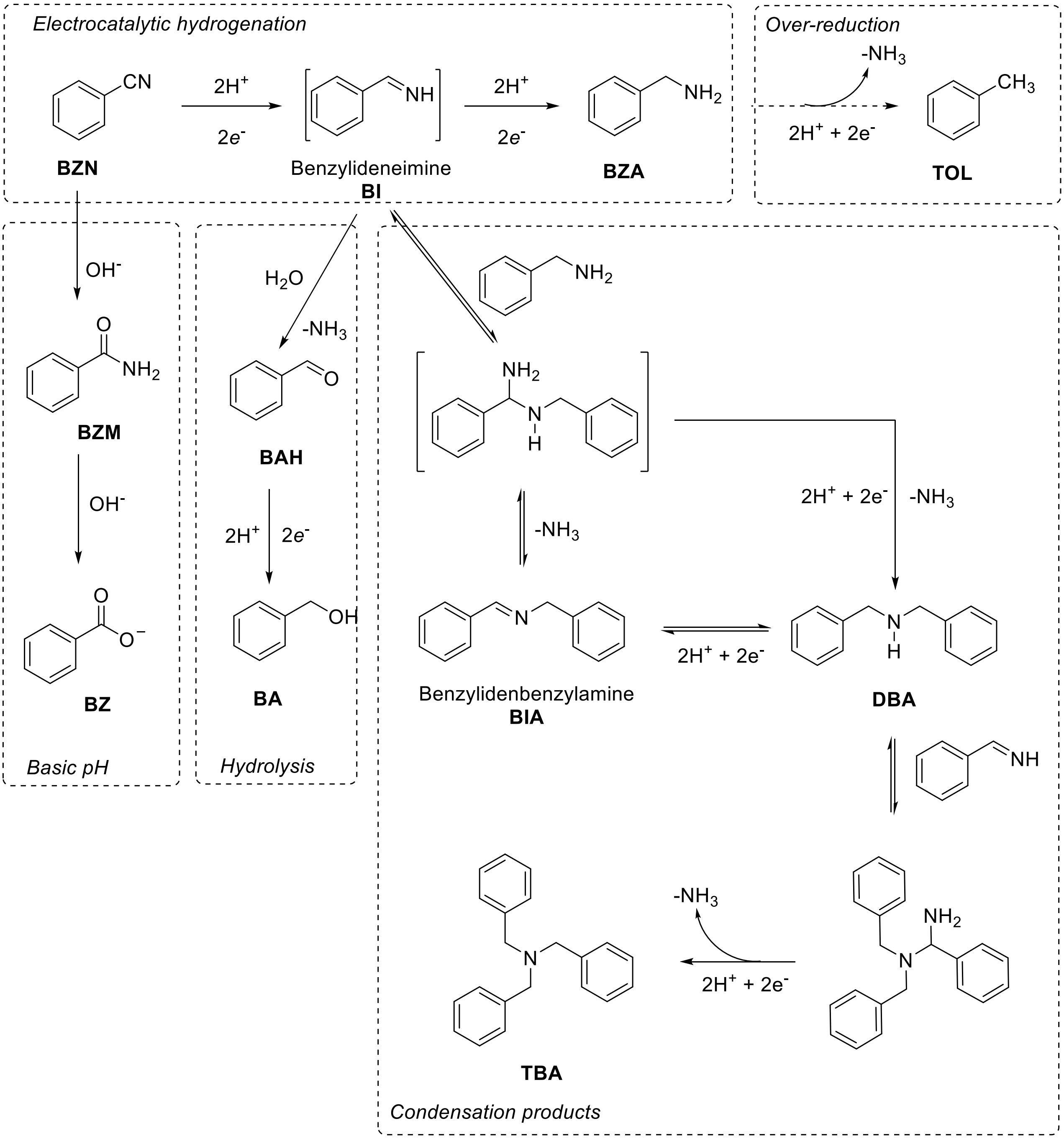

Figure S4. Possible reaction pathways and products in electroreduction of BZN.[1]

S4. Cyclic voltammetry experiments at different BZN concentrations.

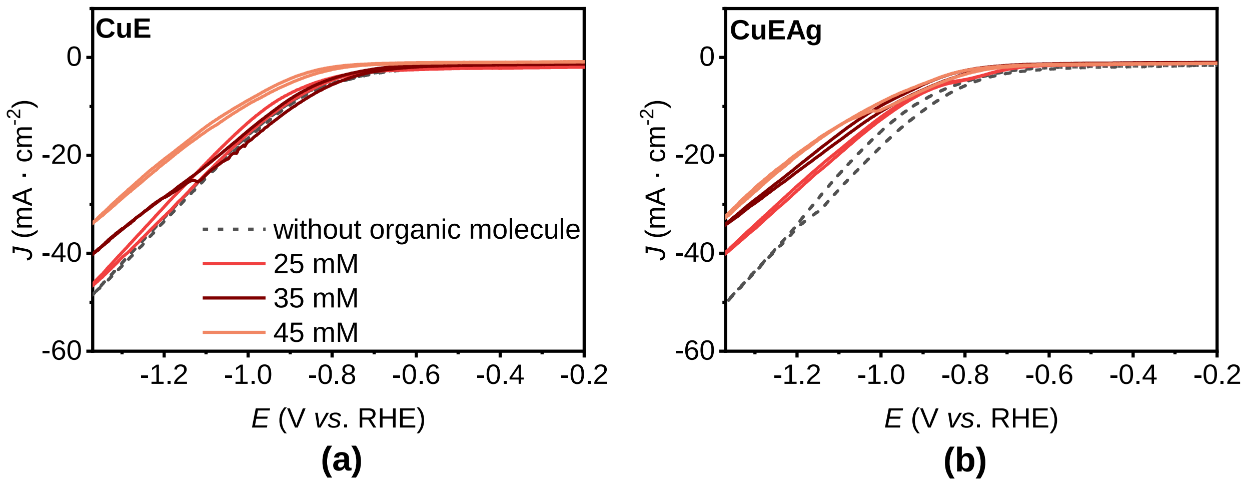

Figure S5. Cyclic voltammetry at different BZN concentration with **(a)** CuE and **(b)** CuEAg. The legend in **(a)** also applies for **(b)**.

S5. Electrochemical active surface area of CuE and CuEAg electrodes.

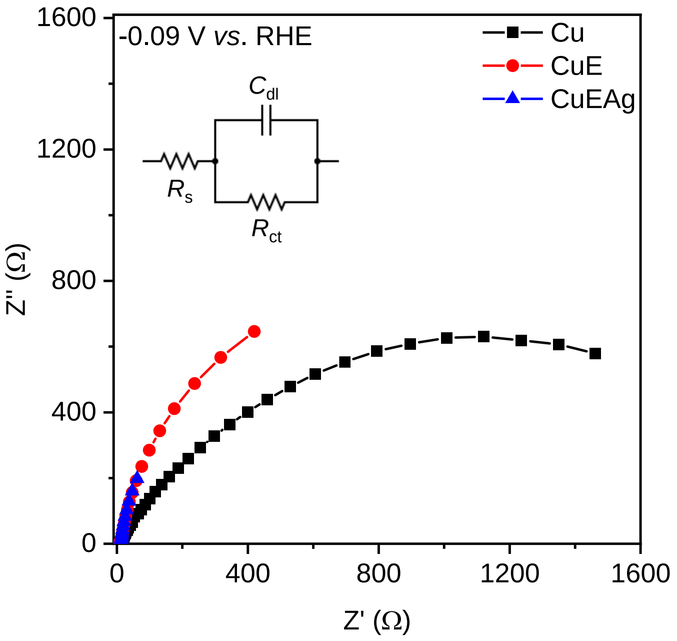

Figure S6. Nyquist plots for the electrodes Cu foil, CuE and CuEAg in 25 mM BZN 0.5 M KCl 70:30 H_2O:CH_3CN at -0.09 V *vs.* RHE, in the frequency range 100 Hz and 100 kHz. This potential fall in a potential region in which no Faradaic processes are observed. The inset shows the simplified Randles circuit use for data fitting in all cases.

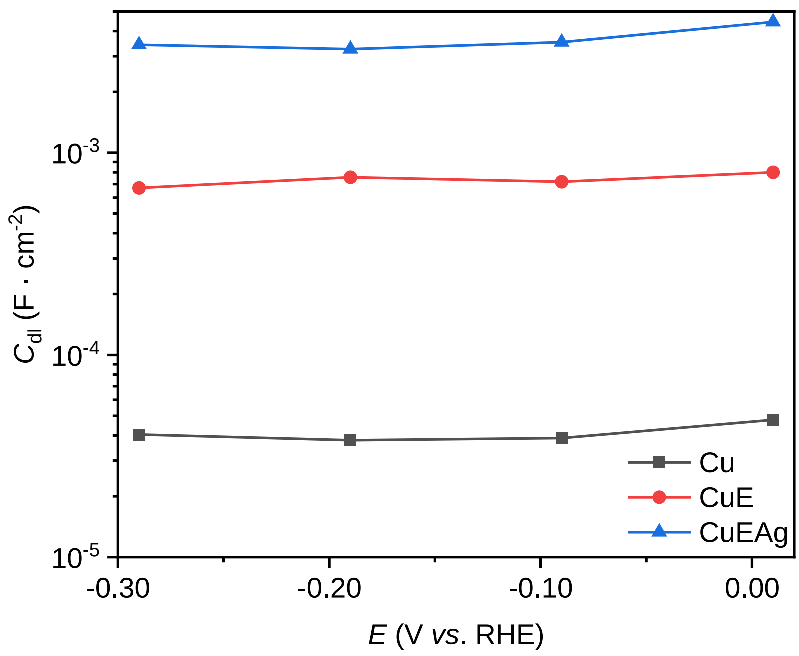

Figure S7. Results of the double-layer capacitance (Cdl) obtained for three electrodes for the BZN electrohydrogenation reaction. Cu foil was used for comparison with the electrodeposited electrodes.

S6. Product Separation and Quantification with HPLC.

Table S1. Retention times and UV maximum absorption wavelength (λ_{max}) for the BZN reduction products and main by-products from the HPLC separation method.

Name	Structure	Retention time (min)	λ_{max} (nm)
Benzylamine (BZA)	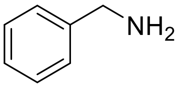	1.90	210
Benzamide (BZM)	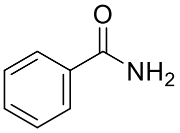	2.76	210
Dibenzylamine (DBA)	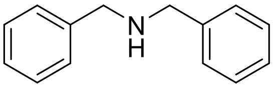	3.00	210
Benzoic Acid (BZ)	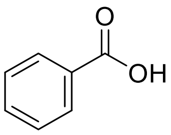	5.25	230
Benzaldehyde (BAH)	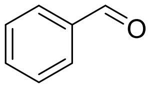	8.01	210
Benzonitrile (BZN)	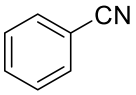	9.89	230

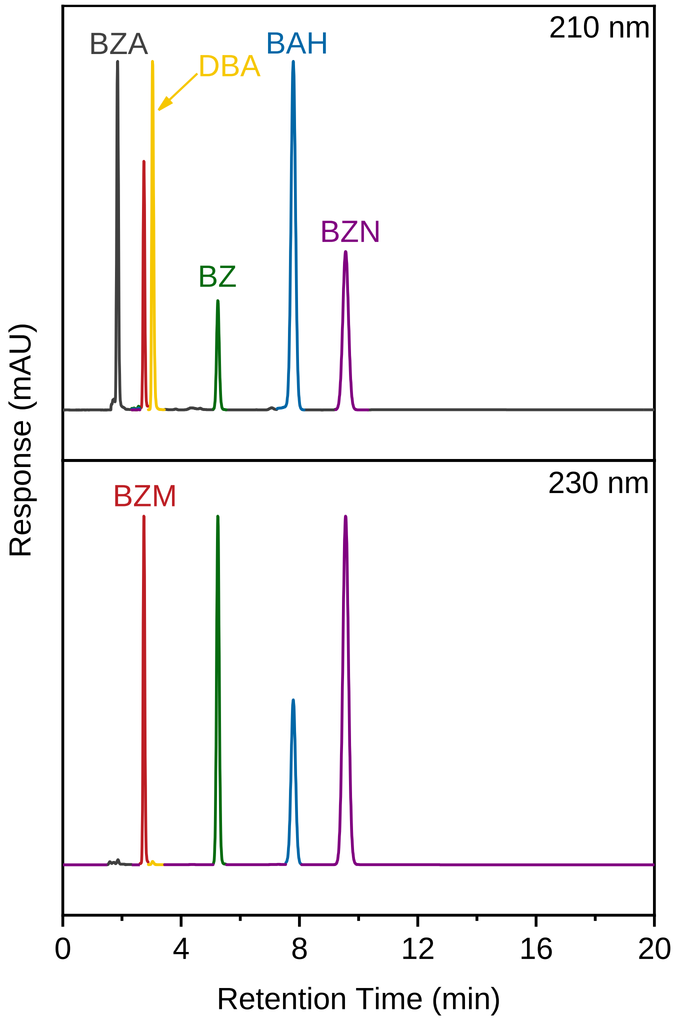

Figure S8. HPLC chromatogram of a 25 mM standard solution each of BZN, BZA, BZM, BZ, BAH and DBA in 0.5 M KCl 70:30 H_2O:CH_3CN.

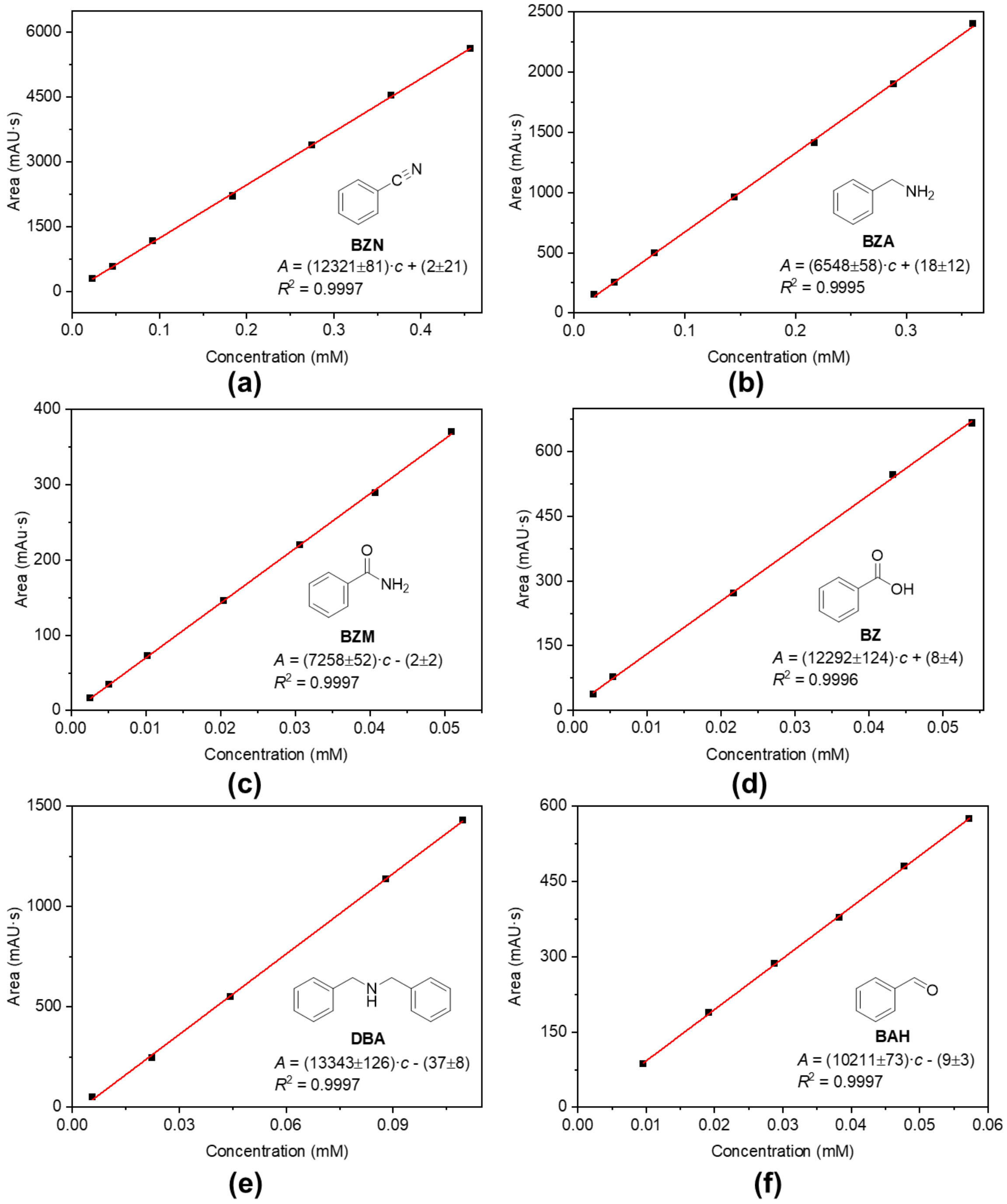

Figure S9. Calibration curves of **(a)** BZN, **(b)** BZA, **(c)** BZM, **(d)** BZ, **(e)** DBA and **(f)** BAH.

Table S2. Retention times and UV maximum absorption wavelength (λ_{max}) for the aromatic nitriles reduction reaction scope from the HPLC separation method.

**Name**	**Structure**	**Retention time (min)**	**λ_{max} (nm)**
2-Methoxybenzonitrile **2a**	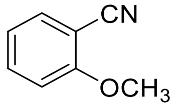	11.33	210
2-Methoxybenzylamine **2b**	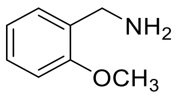	2.23	210
3-Methoxybenzonitrile **3a**	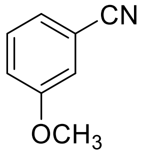	14.19	210
3-Methoxybenzylamine **3b**	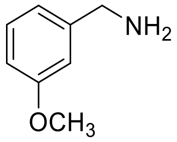	1.85	210
4-Methoxybenzonitrile **4a**	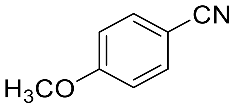	11.84	250
4-Methoxybenzylamine **4b**	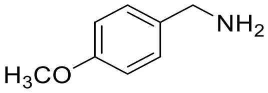	1.85	230
4-Fluorobenzonitrile **5a**	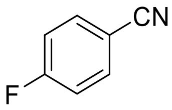	11.09	230
4-Fluorobenzylamine **5b**	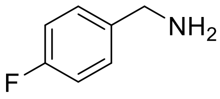	1.88	210
4-(Methylsulfonyl)benzonitrile **6a**	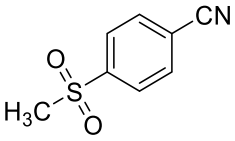	4.77	230
4-(Methylsulfonyl)benzylamine **6b**	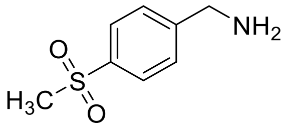	1.78	210
4-Aminobenzonitrile **7a**	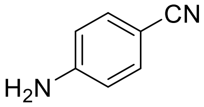	4.51	280
4-(Aminomethyl)aniline **7b**	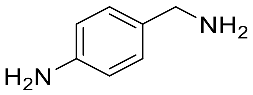	1.62	210

Benzylcyanide **8a**	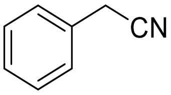	10.59	210
Phenethylamine **8b**	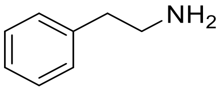	1.90	210
3-Phenylpropionitrile **9a**	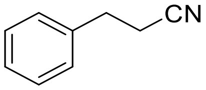	14.30	210
3-Phenylpropylamine **9b**	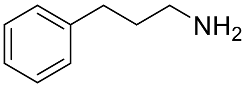	1.87	210

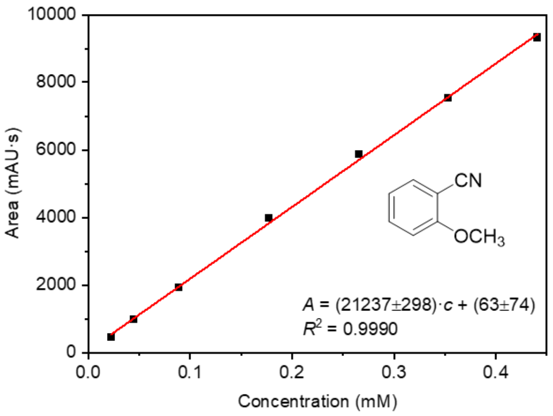

Area (mAU·s)
Concentration (mM)
CN
OCH3
A = (21237±298)·c + (63±74)
R2 = 0.9990

(a)

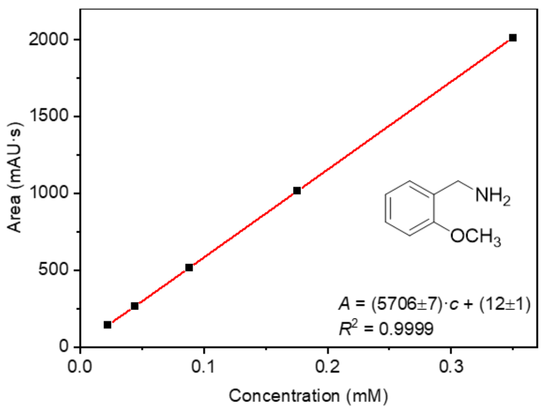

Area (mAU·s)
Concentration (mM)
NH2
OCH3
A = (5706±7)·c + (12±1)
R2 = 0.9999

(b)

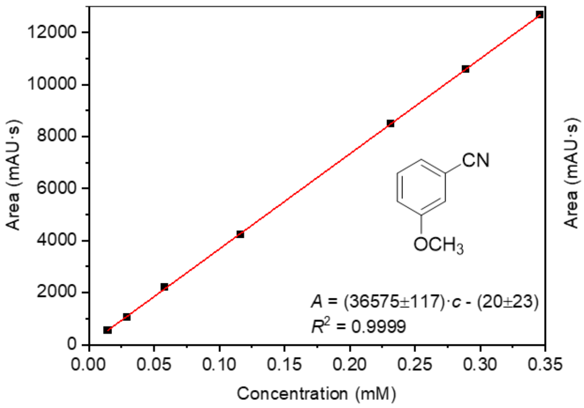

Area (mAU·s)
Concentration (mM)
CN
OCH3
A = (36575±117)·c - (20±23)
R2 = 0.9999

(c)

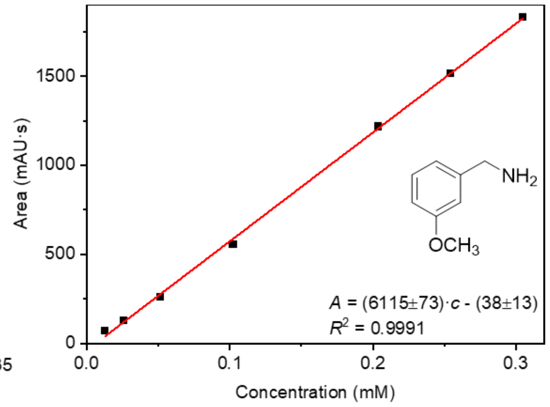

Area (mAU·s)
Concentration (mM)
NH2
OCH3
A = (6115±73)·c - (38±13)
R2 = 0.9991

(d)

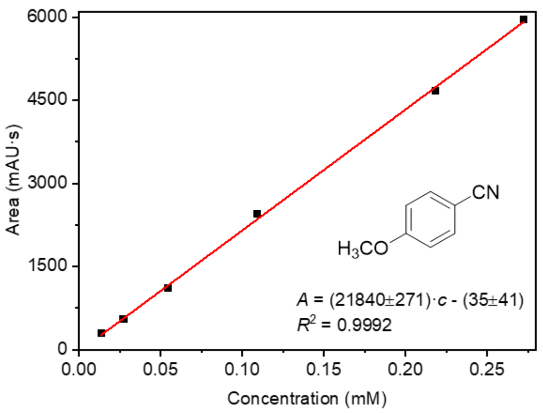

Area (mAU·s)
Concentration (mM)
CN
H3CO
A = (21840±271)·c - (35±41)
R2 = 0.9992

(e)

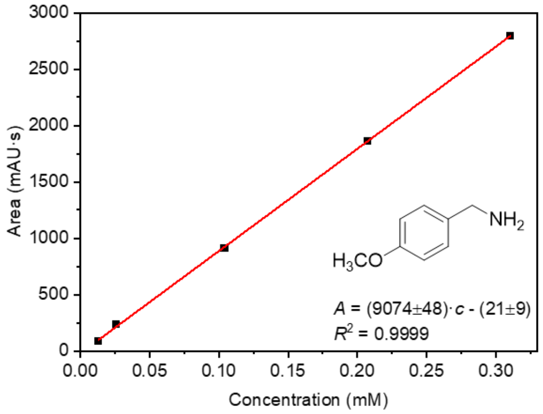

Area (mAU·s)
Concentration (mM)
NH2
H3CO
A = (9074±48)·c - (21±9)
R2 = 0.9999

(f)

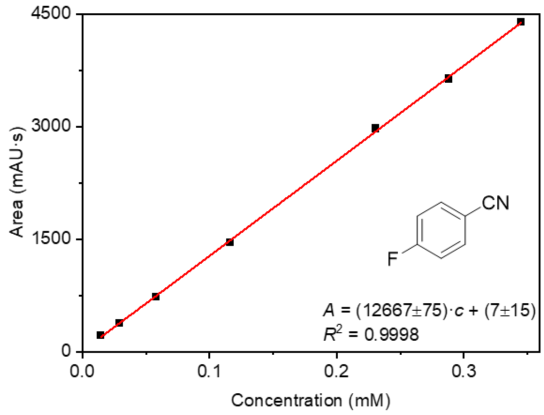

Area (mAU·s)
Concentration (mM)
CN
F
A = (12667±75)·c + (7±15)
R2 = 0.9998

(g)

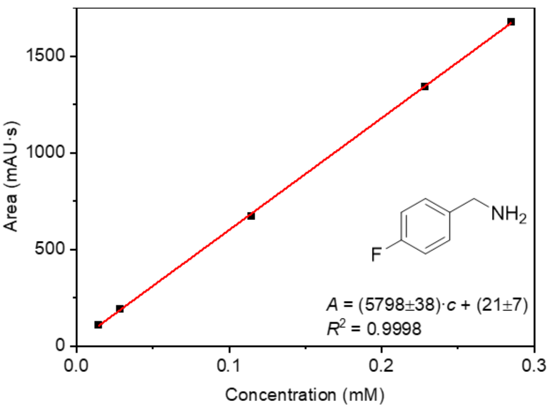

Area (mAU·s)
Concentration (mM)
NH2
F
A = (5798±38)·c + (21±7)
R2 = 0.9998

(h)

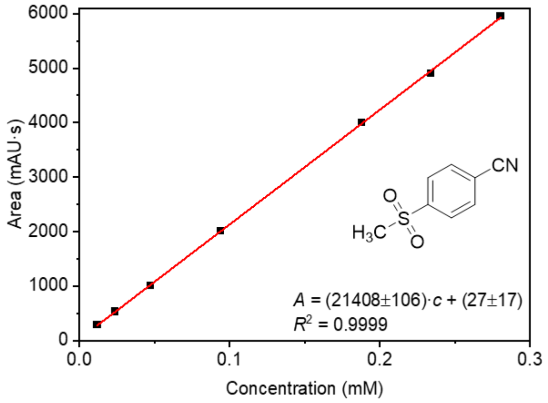
Area (mAU·s)
Concentration (mM)
CN
O
S
H3C
O
A = (21408±106)·c + (27±17)
R^2 = 0.9999

(i)

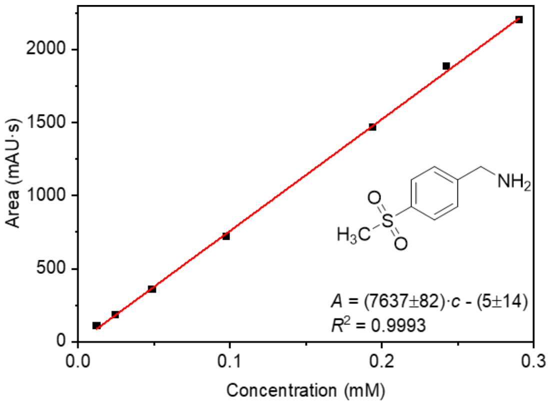
Area (mAU·s)
Concentration (mM)
NH2
O
S
H3C
O
A = (7637±82)·c - (5±14)
R^2 = 0.9993

(j)

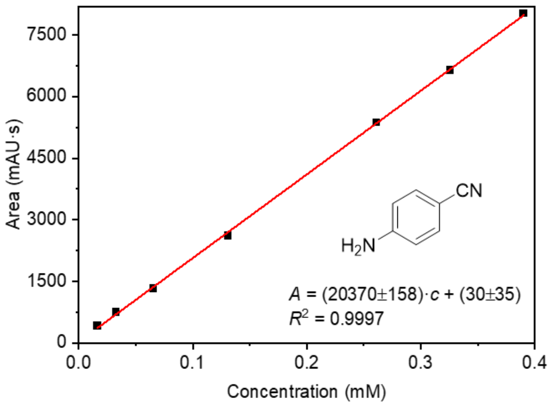
Area (mAU·s)
Concentration (mM)
CN
H2N
A = (20370±158)·c + (30±35)
R^2 = 0.9997

(k)

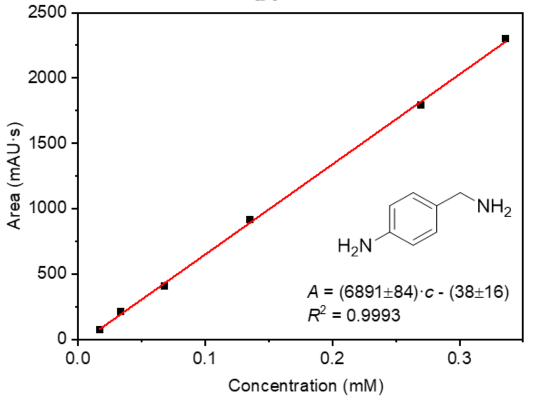
Area (mAU·s)
Concentration (mM)
NH2
H2N
A = (6891±84)·c - (38±16)
R^2 = 0.9993

(l)

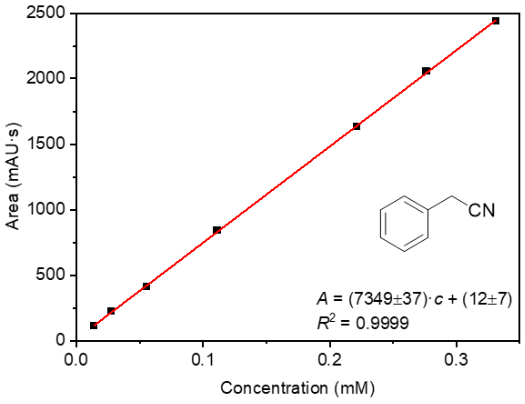
Area (mAU·s)
Concentration (mM)
CN
A = (7349±37)·c + (12±7)
R^2 = 0.9999

(m)

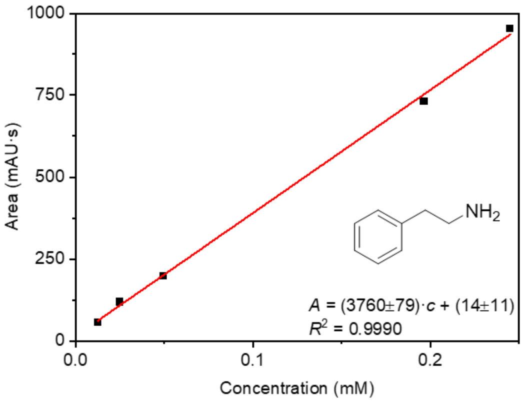
Area (mAU·s)
Concentration (mM)
NH2
A = (3760±79)·c + (14±11)
R^2 = 0.9990

(n)

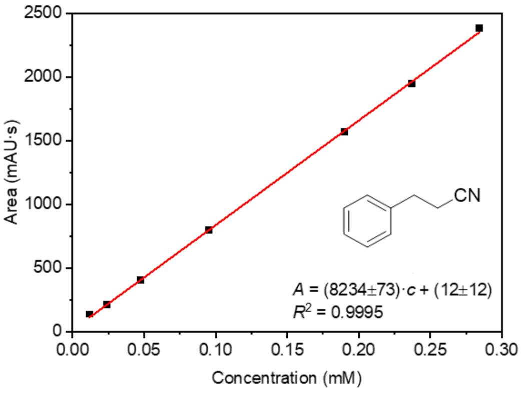
Area (mAU·s)
Concentration (mM)
CN
A = (8234±73)·c + (12±12)
R^2 = 0.9995

(o)

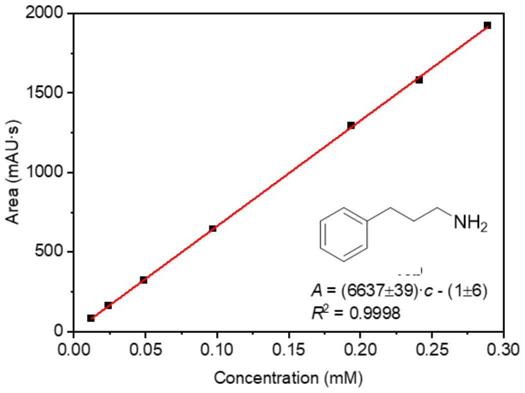
Area (mAU·s)
Concentration (mM)
NH2
A = (6637±39)·c - (1±6)
R^2 = 0.9998

(p)

Figure S10. Calibrations curves for the aromatic nitriles electroreduction reaction scope obtained from the HPLC analysis: **(a)** 2-Methoxybenzonitrile **2a**, **(b)** 2-Methoxybenzylamine **2b**, **(c)** 3-Methoxybenzonitrile **3a**, **(d)** 3-Methoxybenzylamine **3b**, **(e)** 4-Methoxybenzonitrile **4a**, **(f)** 4-Methoxybenzylamine **4b**, **(g)** 4-Fluorobenzonitrile **5a**, **(h)** 4-Fluorobenzylamine **5b**, **(i)** 4-(Methylsulfonyl)benzonitrile **6a**, **(j)** 4-(Methylsulfonyl)benzylamine **6b**, **(k)** 4-Aminobenzonitrile **7a**, **(l)** 4-(Aminomethyl)aniline **7b**, **(m)** Benzylcyanide **8a**, **(n)** Phenethylamine **8b**, **(o)** 3-Phenylpropionitrile **9a** and **(p)** 3-Phenylpropylamine **9b**.

S7. Benzonitrile Electroreduction Reaction Optimization.

Table S3. Effect of the co-solvent on the electrohydrogenation of BZN using CuE as electrode.

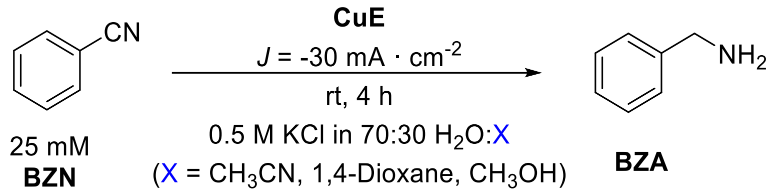

Entry	E_{obs} (V vs. RHE)	Co-solvent	Conversion BZN (%)	Yield (%) BZA	BZM	BAH	FE (%) BZA
1	-1.08	CH_3CN	89	81	2	1	23
2	-1.17	1,4-Dioxane	29	22	3	4	5
3	-1.25	CH_3OH	50	43	2	2	13

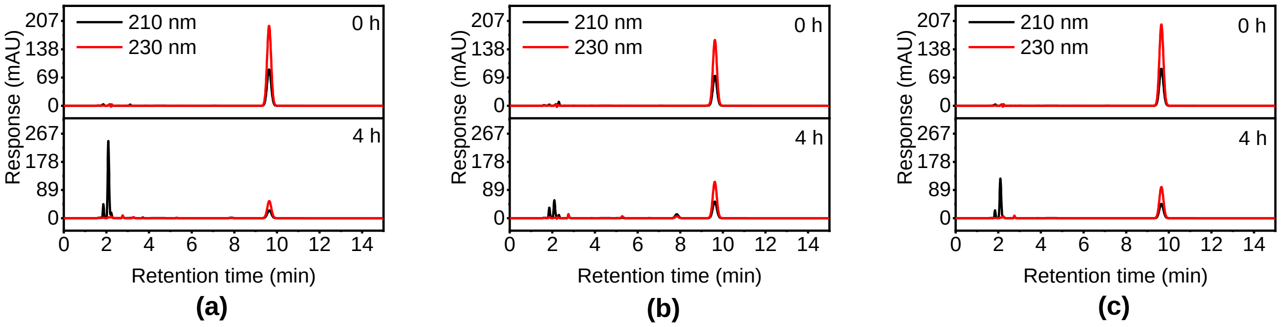

Figure S11. HPLC chromatograms on the electrohydrogenation of BZN for the co-solvent effect: **(a)** CH_3CN, **(b)** 1,4-Dioxane and **(c)** CH_3OH.

Table S4. Effect of the current density on the electrohydrogenation of BZN using CuE and CuEAg as electrodes.

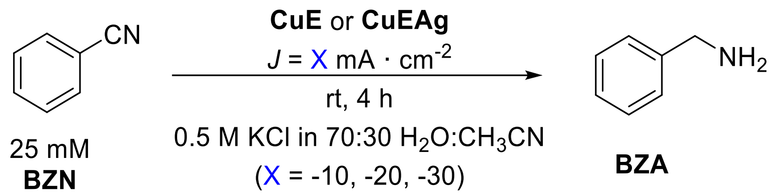

Entry	Electrode	J ($mA{\cdot}cm^{-2}$)	E_{obs} (V *vs.* RHE)	Conversion (%) BZN	Yield (%) BZA	Yield (%) BZM	Yield (%) BAH	Yield (%) BZ	FE (%) BZA
1	CuE	-10	-0.96	58	56	1	0	0	40
2	CuE	-20	-0.94	85	80	2	0	0	35
3	CuE	-30	-1.08	89	81	2	1	1	23
4	CuEAg	-10	-0.82	26	22	2	0	0	24
5	CuEAg	-20	-1.01	87<br>89±4	78<br>79±1	2	2	1	46<br>45±1
6	CuEAg	-30	-1.07	97	87	3	2	0	26
7	Ag	-20	-0.93	8	0	4	0	0	0

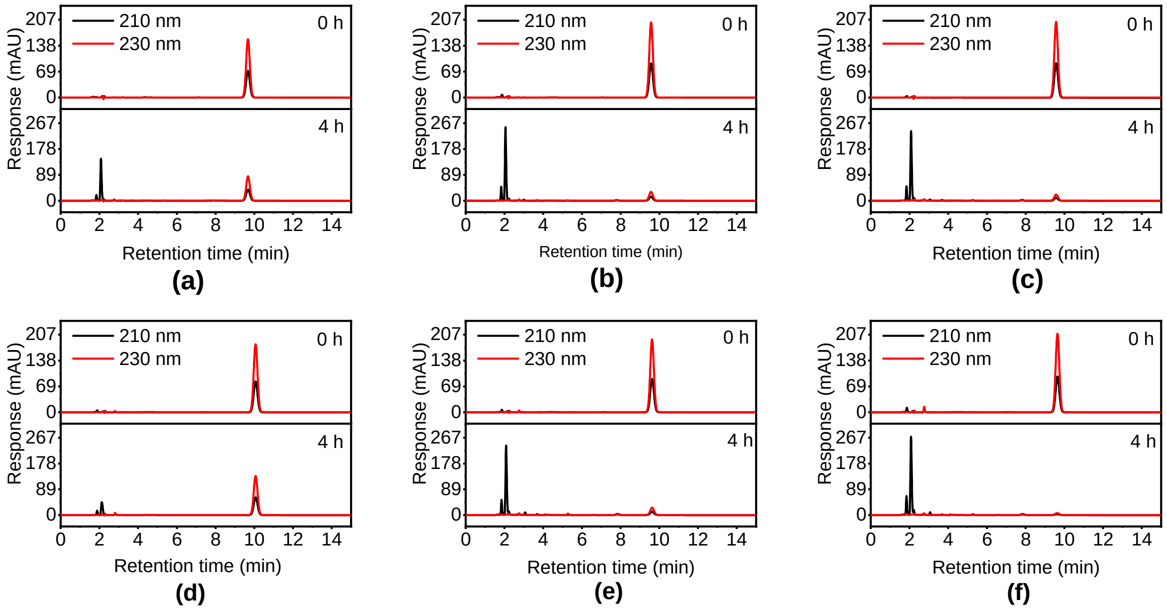

Figure S12. HPLC chromatograms on the electrohydrogenation of BZN for the current density effect: **(a)**, **(d)** -10 mA · cm^{-2}; **(b)**, **(e)** -20 mA · cm^{-2} and **(c)**, **(f)** -30 mA · cm^{-2}, using **(a-c)** CuE and **(d-f)** CuEAg as electrodes

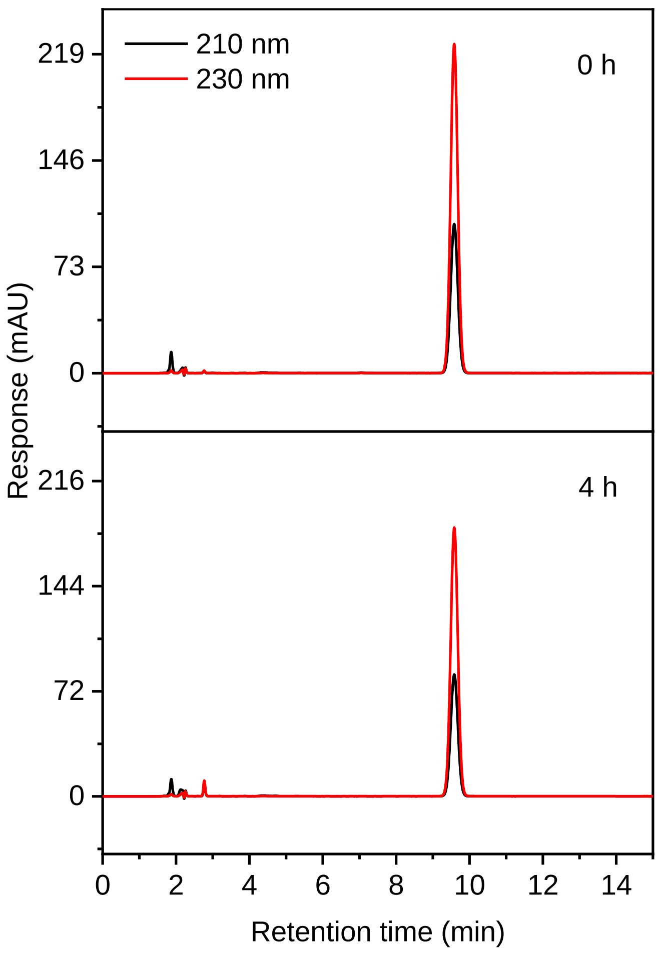

Figure S13. HPLC chromatogram on the electrohydrogenation of BZN using Ag foil as electrode.

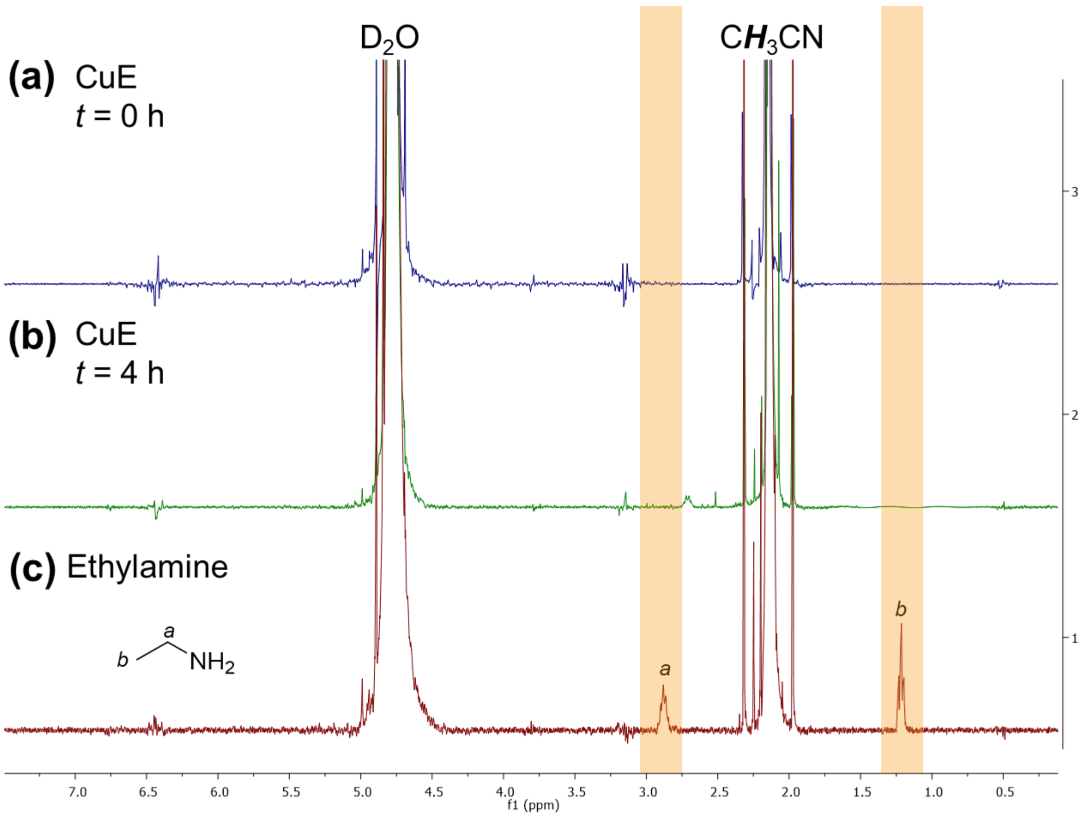

Figure S14. ^{1}H-NMR spectra of the electrolyte (70:30 H_2O:CH_3CN, without benzonitrile) using CuE as WE **(a)** before (0 h) and **(b)** after 4 h of electrolysis applying -20 mA·cm^{-2}. **(c)** Reference spectrum of ethylamine, prepared at 25 mM concentration in 70:30 H_2O:CH_3CN is included for comparison. No signals corresponding to ethylamine or other CH_3CN reduction products were observed.

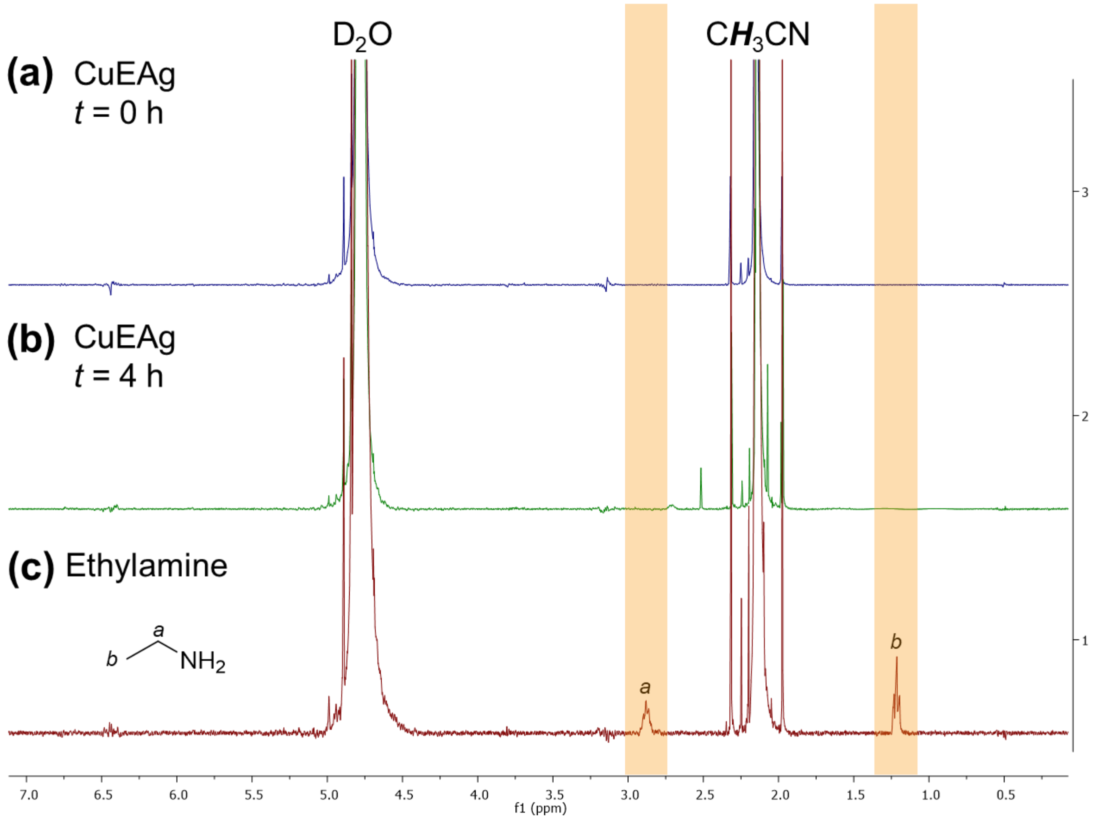

Figure S15. ^{1}H-NMR spectra of the electrolyte (70:30 H_2O:CH_3CN, without benzonitrile) using CuEAg as WE **(a)** before (0 h) and **(b)** after 4 h of electrolysis applying -20 mA·cm^{-2}. **(c)** Reference spectrum of ethylamine, prepared at 25 mM concentration in 70:30 H_2O:CH_3CN is included for comparison. No signals corresponding to ethylamine or other CH_3CN reduction products were observed.

Table S5. Effect of the electrolyte type on the electrohydrogenation of BZN using CuE and CuEAg as electrodes.

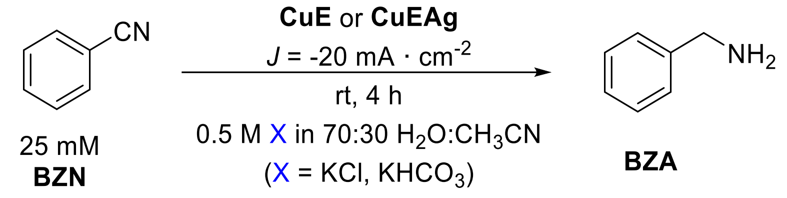

Entry	Electrode	Electrolyte	E_{obs} (V *vs.* RHE)	Conversion (%) BZN	Yield (%) BZA	Yield (%) BZM	Yield (%) BAH	Yield (%) BZ	FE (%) BZA
1	CuE	KCl	-0.94	85	80	2	0	0	35
2	CuE	$KHCO_3$	-1.03	78	73	1	1	1	21
3	CuEAg	KCl	-1.01	87<br>89±4	78<br>79±1	2	2	1	46<br>45±1
4	CuEAg	$KHCO_3$	-1.05	83	72	1	1	1	33

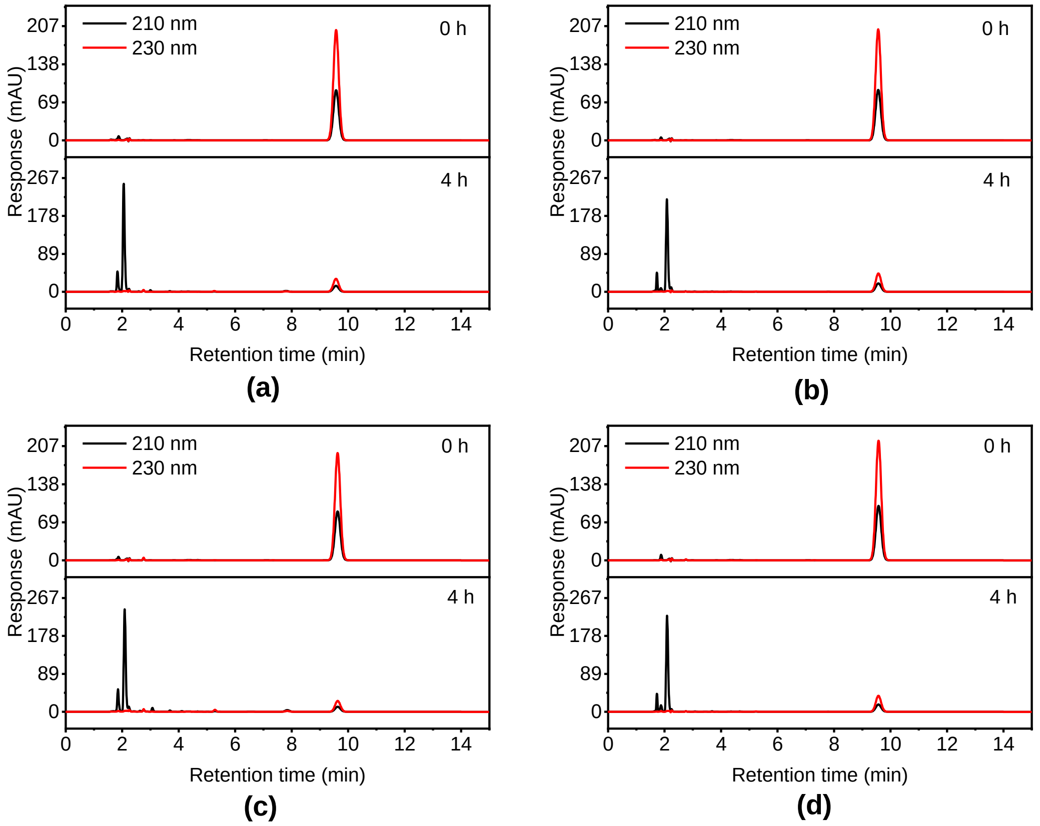

Figure S16. HPLC chromatograms on the electrohydrogenation of BZN for the electrolyte type: **(a)**, **(c)** 0.5 M KCl and **(b)**, **(d)** 0.5 M $KHCO_3$ using **(a-b)** CuE and **(c-d)** CuEAg as electrodes.

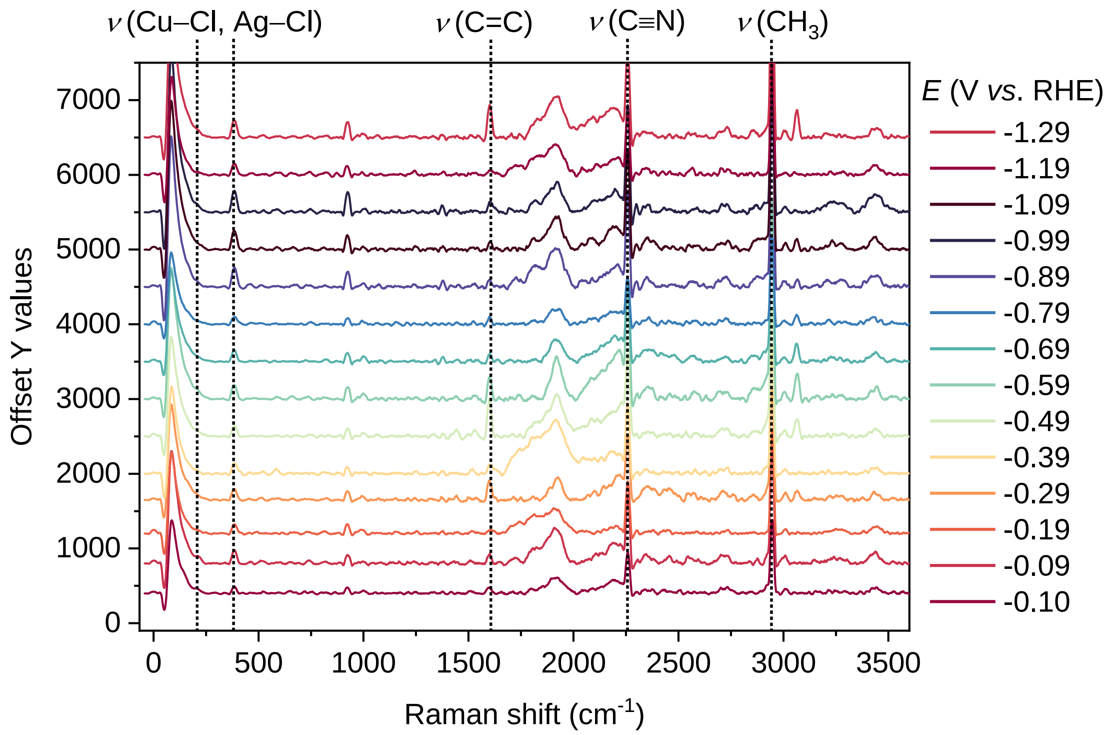

Figure S17. Raman spectra at different applied potentials using CuEAg as WE and 25 mM BZN in 0.5 M KCl as electrolyte in a 70:30 H_2O:CH_3CN mixture.

Table S6. Effect of electrolyte concentration on the electrohydrogenation of BZN using CuE and CuEAg as electrodes.

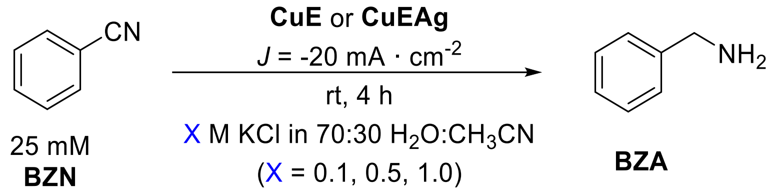

Entry	Electrode	[KCl] (M)	E_{obs} (V *vs.* RHE)	Conversion (%) BZN	Yield (%) BZA	BZM	BAH	BZ	FE (%) BZA
1	CuE	0.1	-1.46	85	75	1	2	0	23
2		0.5	-0.94	85	80	2	0	0	35
3		1.0	-1.11	80	70	2	1	0	11
4	CuEAg	0.1	-1.29	90	75	3	3	3	33
5		0.5	-1.01	87<br>89±4	78<br>79±1	2	2	1	46<br>45±1
6		1.0	-1.06	88	77	2	2	1	26

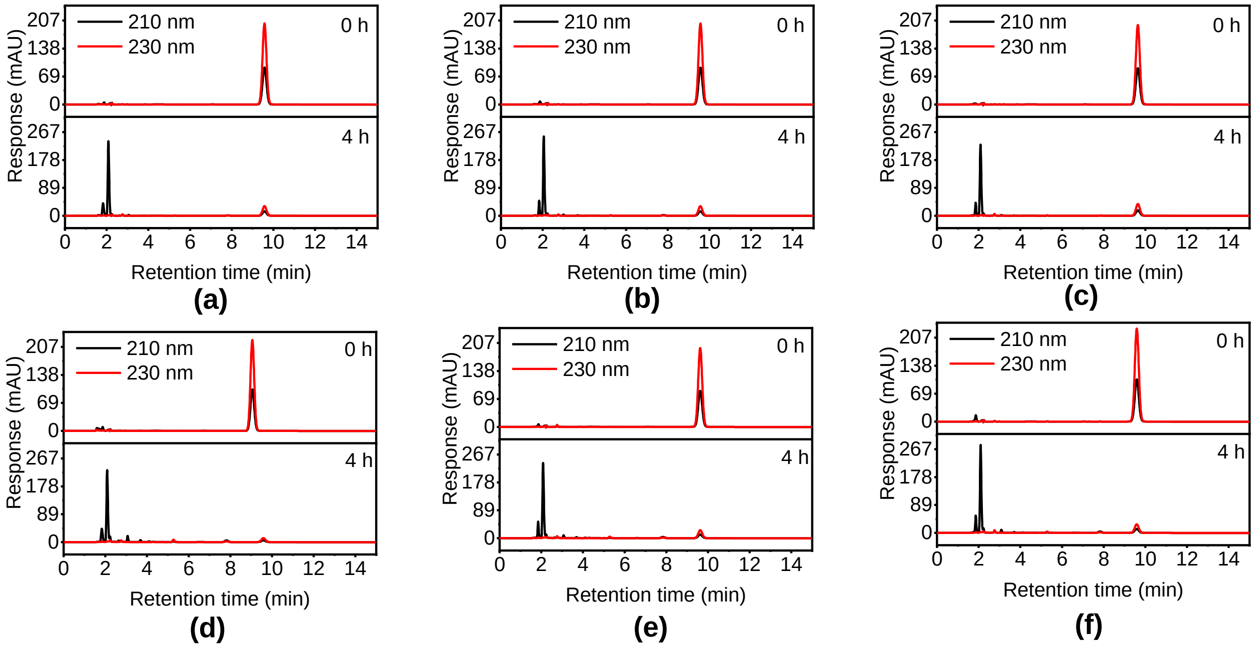

Figure S18. HPLC chromatograms on the electrohydrogenation of BZN for the KCl electrolyte concentration: **(a)**, **(d)** 0.1 M; **(b)**, **(e)** 0.5 M; and **(c)**, **(f)** 1.0 M using **(a-c)** CuE and **(d-f)** CuEAg as electrodes

Table S7. Effect of BZN concentration on the electrohydrogenation of BZN using CuEAg as electrode.

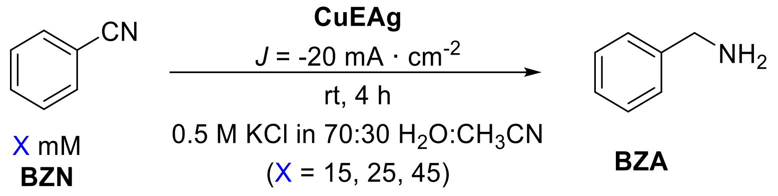

Entry	[BZN] (mM)	E_{obs} (V *vs.* RHE)	Conversion (%) BZN	Yield (%)				FE (%)
				BZA	BZM	BAH	BZ	BZA
1	15	-0.87	95	75	5	4	4	19
2	25	-1.01	87<br>89±4	78<br>79±1	2	2	1	46<br>45±1
3	45	-0.86	87	75	2	2	5	63

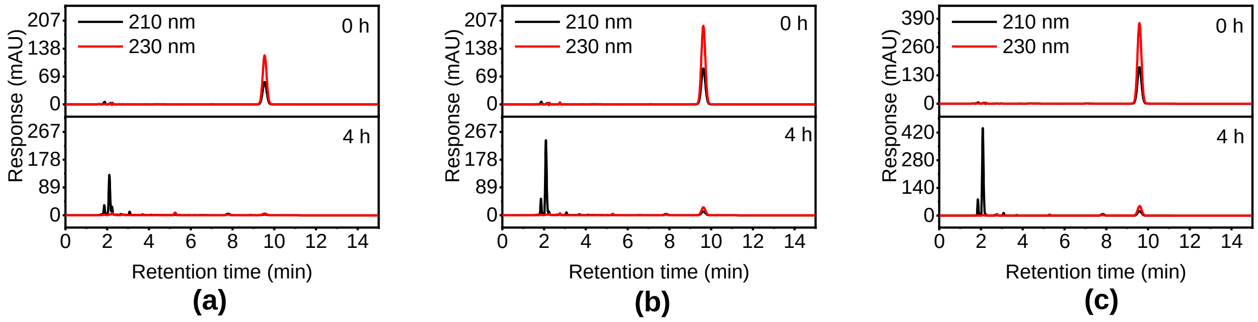

Figure S19. HPLC chromatograms on the electrohydrogenation of BZN for the BZN concentration: **(a)** 15 mM, **(b)** 25 mM and **(c)** 45 mM using CuEAg as electrode

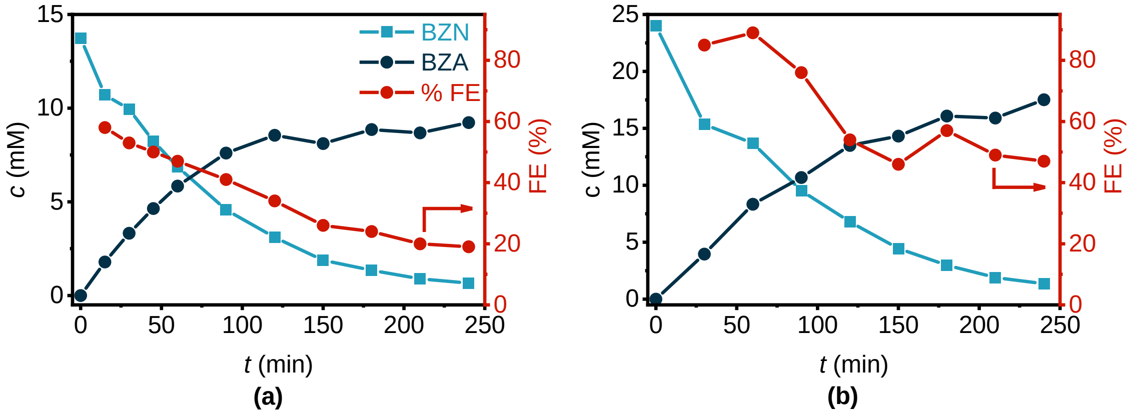

Figure S20. Kinetic profile of the BZN electrohydrogenation reaction using CuEAg as cathode and a constant current density -20 mA·cm^{-2} at different BZN starting concentration: **(a)** 15 mM and **(b)** 25 mM. The legend in **(a)** also applies for **(b)**.

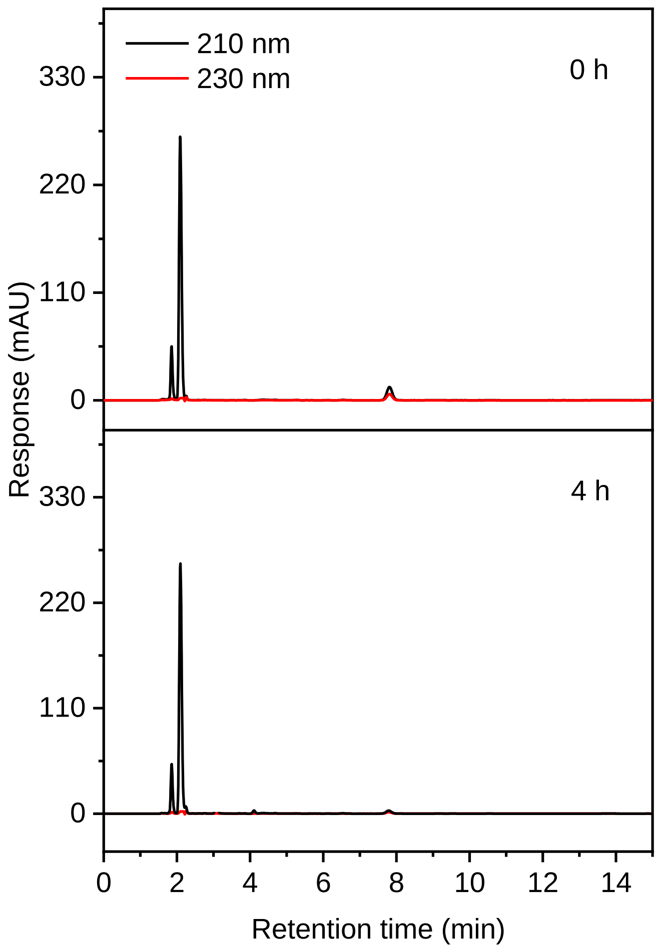

Figure S21. HPLC chromatogram for the electrohydrogenation reaction of 25 mM BZA in 0.5 M KCl, using CuEAg as electrode and applying J = -20 mA · cm^{-2}.

S8. Detection of H_2 from the electrohydrogenation of benzonitrile.

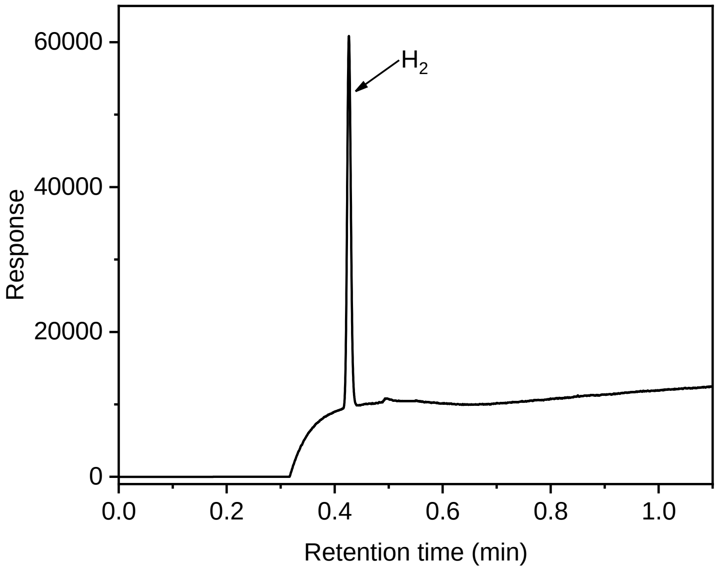

Figure S22. Chromatogram spectra for the detection of H_2 using a gas chromatography (Micro GC 490 Agilent Technologies) of a reaction of 25 mM BZN in 0.5 M KCl 70:30 H_2O:CH_3CN using CuEAg as cathode.

S9. NMR deuteration experiments.

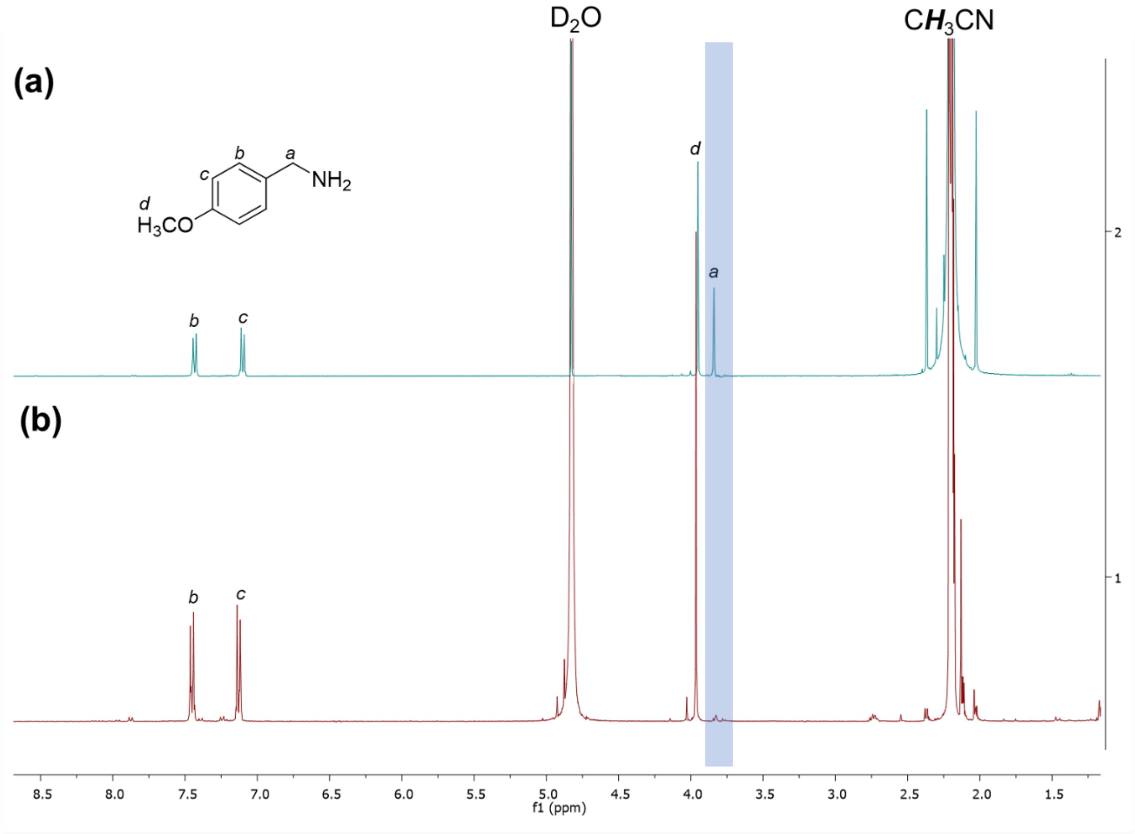

Figure S23. Electrohydrogenation of 4-methoxybenzonitrile **4a** to 4-methoxybenzylamine **4b** in 0.5 M KCl 70:30 D_2O:CH_3CN using CuEAg as electrode and J = -20 mA·cm^{-2}. Showing the disappearance of the deuterated methylene group **(a)** Spectrum of commercial 4-methoxybenzylamine **4b**, **(b)** Electrohydrogenation reaction after t = 6 h. ^{1}H-NMR (400 MHz, D_2O) δ (ppm) 3.77 (*s*, H_a), 7.69 (*d*, H_b), 7.37 (*d*, H_c), 4.20 (*s*, H_d). Code: *s* singlet, *d* doublet.

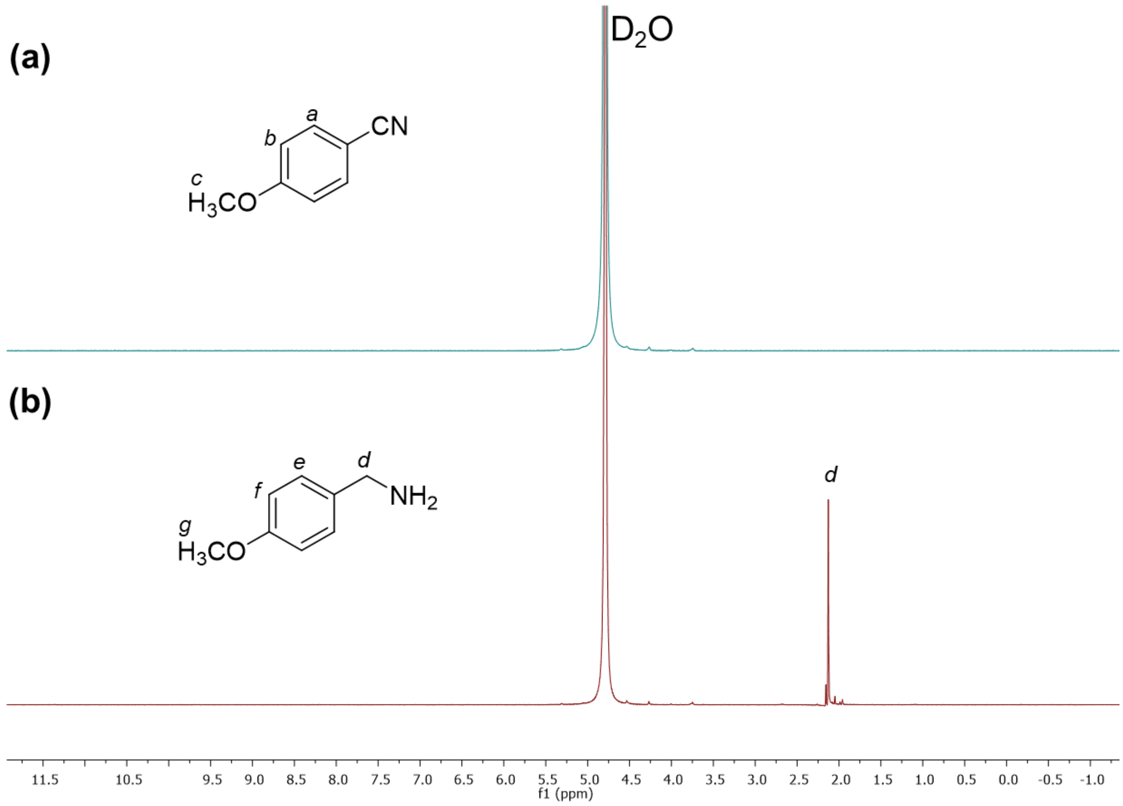

Figure S24. Electrohydrogenation of 4-methoxybenzonitrile **4a** to 4-methoxybenzylamine **4b** in 0.5 M KCl 70:30 D_2O:CH_3CN using CuEAg as electrode and J = -20 mA·cm^{-2} **(a)** t = 0 h, **(b)** t = 6 h. ^{2}H-NMR (500 MHz, D_2O) δ (ppm) 2.13 (s, H_d). Code: *s* singlet.

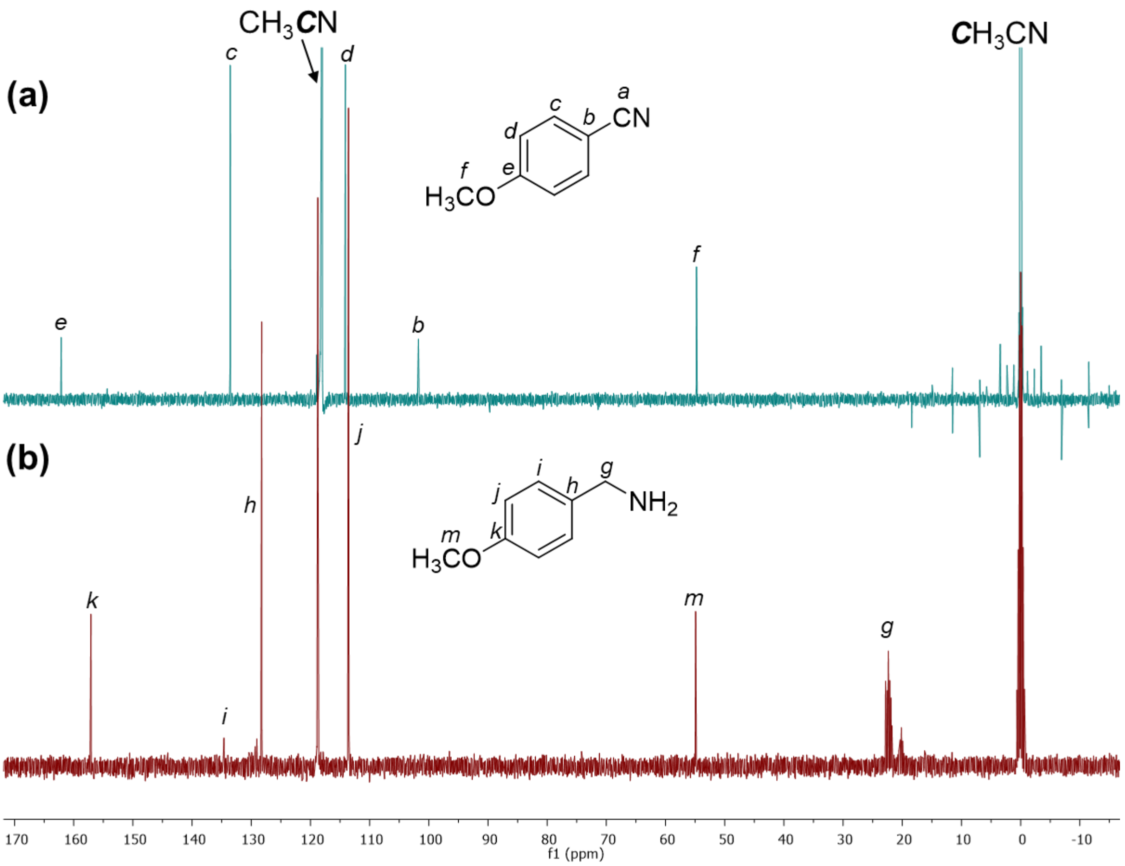

Figure S25. Electrohydrogenation of 4-methoxybenzonitrile **4a** to 4-methoxybenzylamine **4b** in 0.5 M KCl 70:30 D_2O:CH_3CN using CuEAg as electrode and J = -20 mA·cm^{-2} **(a)** t = 0 h, **(b)** t = 6 h. ^{13}C-NMR (400 MHz, D_2O) δ (ppm) **(a)** 162.1 (C_e, *s*), 133.5 (C_c, *s*), 114.1 (C_d, *s*), 101.8 (C_b, *s*), 54.8 (C_f, *s*), **(b)** 157.1 (C_k, *s*), 134.0 (C_i, *s*), 128.2 (C_h, *s*), 113.6 (C_j, *s*), 54.9 (C_m, *s*), 22.2 (C_g, *m*). Code: *s* singlet, *m* multiplet.

S10. Electrohydrogenation Reaction Scope Results.

Table S8. Results of conversion, yield and faradaic efficienty (FE) for the electrohydrogenation of aromatic nitriles scope.

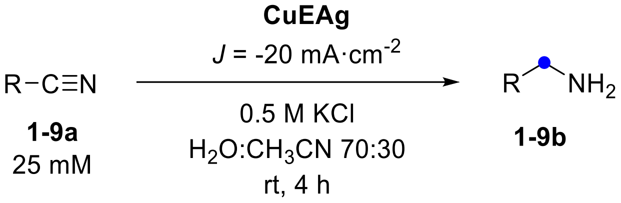

Entry	Nitrile	Amine	E_{obs} (V *vs.* RHE)	Conversion (%)	Yield (%)	FE (%)
1	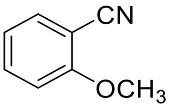 **2a**	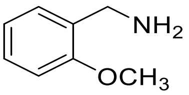 **2b**	-0.94	29	29	17
2	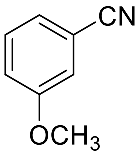 **3a**	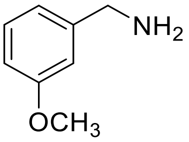 **3b**	-0.96	58	56	34
3	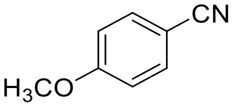 **4a**	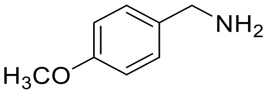 **4b**	-1.00	68	67	42
4	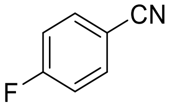 **5a**	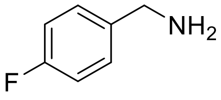 **5b**	-1.03	79	72	34
5	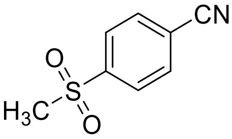 **6a**	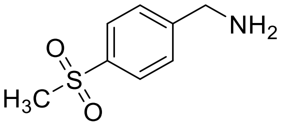 **6b**	-1.01	93	87	38
6	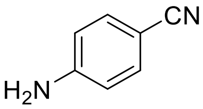 **7a**	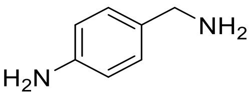 **7b**	-1.02	38	38	24

7	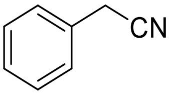 **8a**	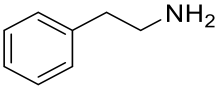 **8b**	-0.92	43	45	21
8	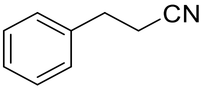 **9a**	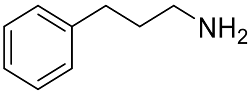 **9b**	-0.91	25	18	6

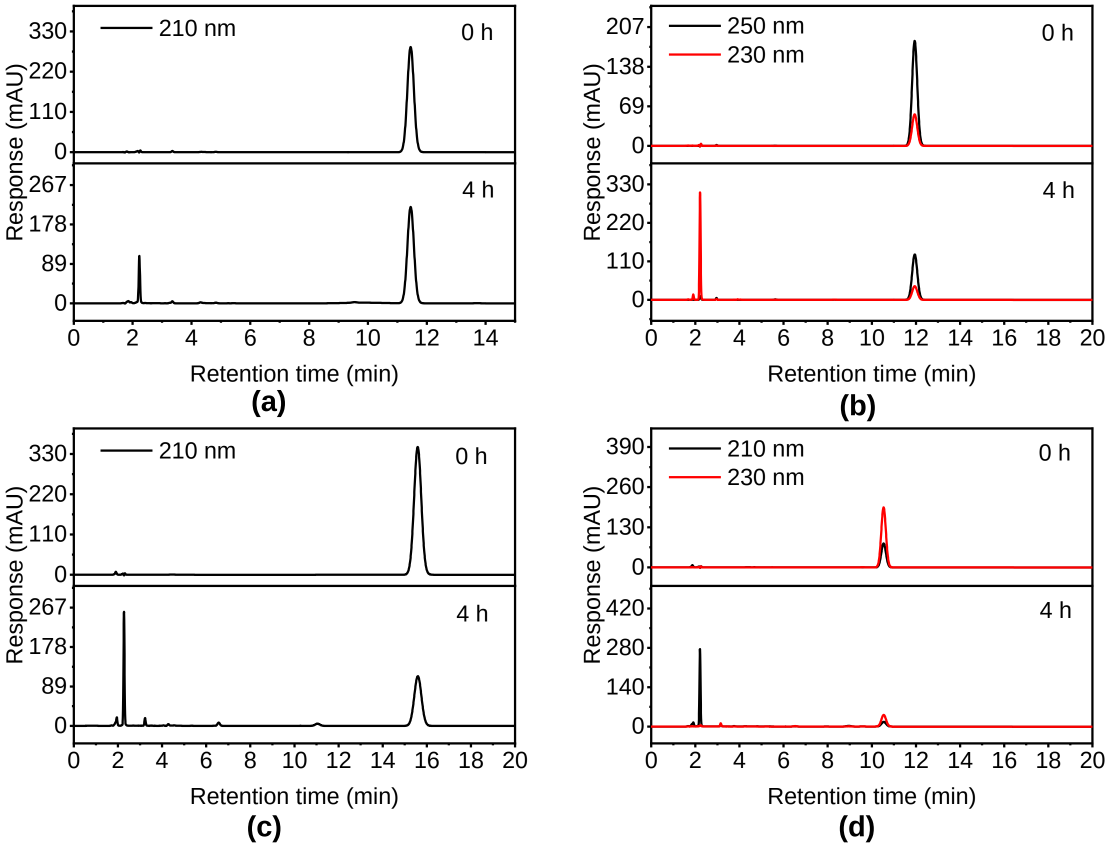

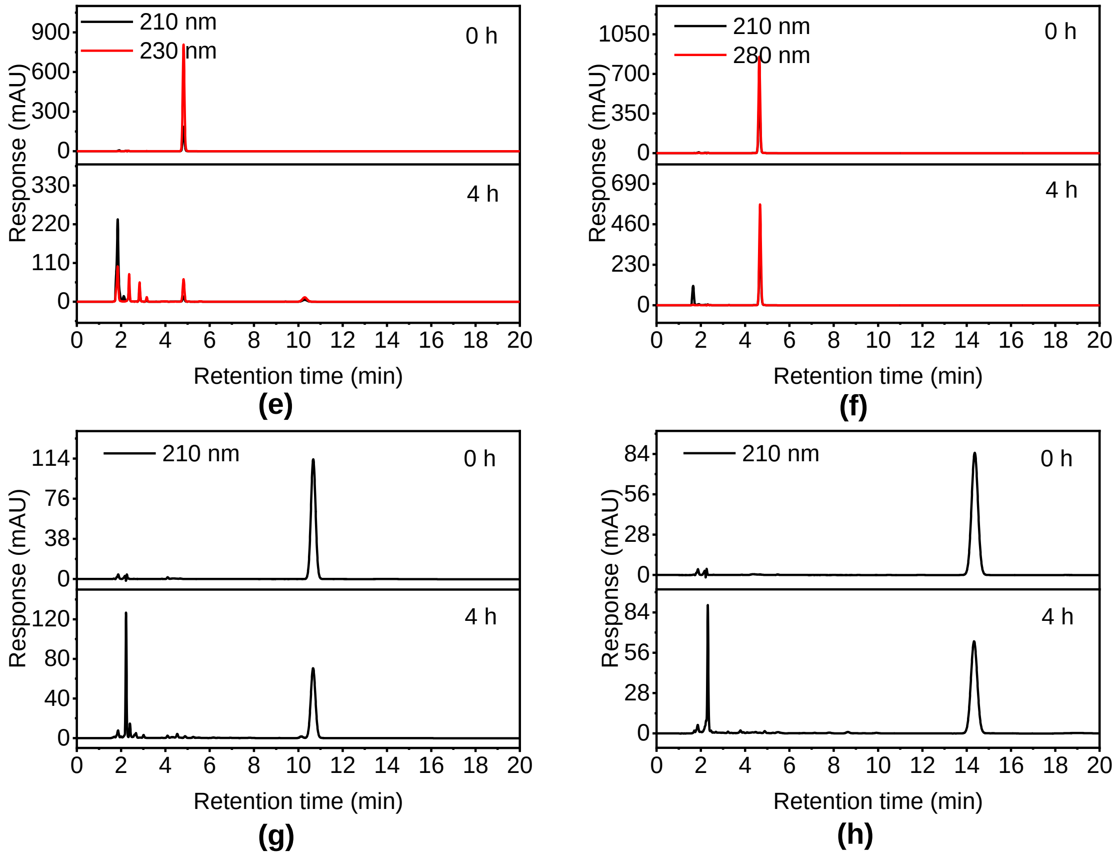

Figure S26. HPLC chromatograms for the aromatic nitriles scope: **(a) 2a**/**2b**, **(b) 3a**/**3b**, **(c) 4a**/**4b**, **(d) 5a**/**5b**, **(e) 6a**/**6b**, **(f) 7a**/**7b**, **(g) 8a**/**8b** and **(h) 9a**/**9b**.

S11. Green Chemistry Metrics.

As previously describe by Waldvogel and coworkers[2], to evaluate the sustainability of our electrohydrogenation method, we calculate six different green chemistry metrics. For the evaluation of our method we only consider the reaction, excluding electrocatalyst preparations, work-up procedure, catalyst, among others.

Similar to Waldvogel and coworkers[2] we include the cost only for consumed and non-recoverable chemicals assuming 1 mol of starting material and using the catalogue prices from Sigma-Aldrich (for the Spain market). To determine the price per gram of chemicals we used the largest available package for chemical and solvents are calculated on the base price of 2.5 L.

The economic aspect (Eco), atom economy (AE) and effective mass yield (EMY) were calculated according to equation (S1)-(S3), respectively.[2,3]

$$\text{Eco} = \frac{\text{product value per mol} \cdot \text{chemical yield \%}}{\sum \text{reagent cost per mol}} \cdot 100\% \tag{S1}$$

$$\text{AE} = \frac{\text{molecular mass of desired product}}{\text{molecular mass of all products}} \cdot 100\% \tag{S2}$$

$$\text{EMY} = \frac{\text{mass of desired product}}{\text{mass of non-benign reagents}} \cdot 100\% \tag{S3}$$

The reaction mass efficiency (RME) was calculated as previously reported Constable *et al*,[4] where for a generic reaction A + B → C, RME is expressed according to equation (8), and represent the percentage of mass of reactants that remain in the product.

$$\text{RME} = \frac{\text{mass of product C}}{\text{mass of A} + \text{mass of B}} \cdot 100\% \tag{S4}$$

To assest the safety perspective, we used NFPA 704 Standard System for the Identification of the Hazards of Materials for Emergency Response from the US National Fire Protection Association (NFPA), communly known as "Safety Square" or "Fire

Diamond". The overal NFPA 704 was computed as an average of each number from the Fire Diamond. In general terms, in a scale of 0 to 4, 0 means very safe and 4 very unsafe.

Table S9 shows the evaluation of our electrohydrogenation method of BZN to BZA. The average overall safety aspect obtained was 3.2

Table S9. Evaluation of the reactants in the electrohydrogenation reaction of BZN to BZA.

Compound	CAS	MW (g/mol)	Price (€/g)	Price (€/mol)	NFPA 704 average	Catalogue Specifications
Benzonitrile	100-47-0	103.12	0.099	10.21	5	1 L, ReagentPlus®, 99%
Benzylamine	100-46-9	107.15	0.156	16.72	5	500 g, ReagentPlus®, 99%
Water	7732-18-5	18.02	0.0318	0.57	0	1 L, deionized for synthesis
Acetonitrile	75-05-8	41.05	0.183	7.51	5	2.5 L, ≥99.9%, gradient grade, suitable for HPLC
Potassium chloride	7447-40-7	74.55	0.053	3.95	1	12 kg, ACS reagent, 99.0-100.5%

The economic aspect calculation is shown in equation (S5) considering 1 mol of starting material and a 78% yield of amine, the cost of water was set to 0 €.

$$\text{Eco} = \frac{16.72\text{€} \cdot 0.78\ (\text{BZA})}{[10.21\text{€}\ (\text{BZN}) + 7.51\text{€}\ (\text{CH}_3\text{CN}) + 3.95\text{€}\ (\text{KCl})]} \cdot 100\% = 60 \qquad \text{(S5)}$$

The AE factor obtained is 77 [see calculation in equation (S6)] and as similar as Waldvogel and coworkers[2], is slightly lowered due to the oxygen production in the anode compartment.

$$\text{AE} = \frac{107.15\ \text{g/mol (BZA)}}{107.15\ \text{g/mol (BZA)} + 31.99\ \text{g/mol (O}_2\text{)}} \cdot 100\% = 77 \qquad \text{(S6)}$$

The RME metric [equation (S7)] was obtained considering our reaction conditions of 25 mM BZN (0.37 mmol, 38.67 mg) in 15 mL of solvent and obtaining a 78% yield of BZA (0.29 mmol, 31.34 mg). Water was not considered in the calculation due to being considered a harmless green reagent/solvent.

$$\text{RME} = \frac{31.34\ \text{mg (BZA)}}{38.67\ \text{mg (BZN)}} \cdot 100\% = 81 \tag{S7}$$

The EMY is obtained as shown in equation (S8):

$$\text{EMY} = \frac{107.17\ \text{g/mol (BZA)}}{103.12\ \text{g/mol (BZN)}} \cdot 100\% = 104 \tag{S8}$$

As noted from the summary in Table S10, AE shows a moderately efficient use of the atoms from the reactants in the final product, due to the oxygen evolution on the anode compartment, can be considered as a benign by-product, while the high values of RME and EMY suggest that the electrohydrogenation procedure effectively converts the reactants into the desired product with minimal waste. From the safety point of view, the slightly high value is related to the moderate flammability and health hazard present in organic compounds used in the electrohydrogenation, with the advantage of using water as source of protons, which can be considered a green/harmless solvent and KCl as electrolyte with relatively low toxicity.

Table S10. Green chemistry metrics summary for electrohydrogenation of BZN to BZA.

Metric	Value
Eco	60
AE	77
RME	81
EMY	104
Safety	3.2
Yield	78

S12. References

(1) Krupka, J.; Pasek, J. Nitrile Hydrogenation on Solid Catalysts-New Insights into the Reaction Mechanism. *Curr. Org. Chem.* **2012**, *16*, 988–1004.

(2) Narobe, R.; Perner, M. N.; Gálvez-Vázquez, M. de J.; Kuhwald, C.; Klein, M.; Broekmann, P.; Rösler, S.; Cezanne, B.; Waldvogel, S. R. Practical Electrochemical Hydrogenation of Nitriles at Nickel Foam Cathode. *Green Chem.* **2024**, *26*, 10567-10574. https://doi.org/10.1039/d4gc03446e.

(3) Sprang, F.; Schupp, N.; Kohlpaintner, P. J.; Gooßen, L. J.; Waldvogel, S. R. E-Dakin Reaction: Oxidation of Hydroxybenzaldehydes to Phenols with Electrochemically Generated Peroxodicarbonate as Sustainable Ex-Cell Oxidizer. *Green Chem.* **2024**, *26* (10), 5862–5868. https://doi.org/10.1039/d3gc04597h.

(4) Constable, D. J. C.; Curzons, A. D.; Cunningham, V. L. Metrics to “green” Chemistry - Which Are the Best? *Green Chem.* **2002**, *4* (6), 521–527. https://doi.org/10.1039/b206169b.
